# Using a 360° Virtual Reality or 2D Video to Learn History Taking and Physical Examination Skills for Undergraduate Medical Students: Pilot Randomized Controlled Trial

**DOI:** 10.2196/13124

**Published:** 2021-11-22

**Authors:** Yi-Ping Chao, Hai-Hua Chuang, Li-Jen Hsin, Chung-Jan Kang, Tuan-Jen Fang, Hsueh-Yu Li, Chung-Guei Huang, Terry B J Kuo, Cheryl C H Yang, Hsin-Yih Shyu, Shu-Ling Wang, Liang-Yu Shyu, Li-Ang Lee

**Affiliations:** 1 Department of Computer Science and Information Engineering Graduate Institute of Biomedical Engineering Chang Gung University Taoyuan Taiwan; 2 Department of Neurology Chang Gung Memorial Hospital Linkou Main Branch Taoyuan Taiwan; 3 Department of Family Medicine Chang Gung Memorial Hospital Taipei Branch & Linkou Main Branch Taoyuan Taiwan; 4 College of Medicine Chang Gung University Taoyuan Taiwan; 5 Department of Otorhinolaryngology-Head and Neck Surgery Chang Gung Memorial Hospital Linkou Main Branch Taoyuan Taiwan; 6 Department of Laboratory Medicine Chang Gung Memorial Hospital, Linkou Main Branch Taoyuan Taiwan; 7 Department of Medical Biotechnology and Laboratory Science Graduate Institute of Biomedical Sciences Chang Gung University Taoyuan Taiwan; 8 Institute of Brain Science National Yang Ming Chiao Tung University Taipei Taiwan; 9 Department of Educational Technology Tamkang University New Taipei Taiwan; 10 Center of Teacher Education National Taiwan University of Science and Technology Taipei Taiwan; 11 Department of Biomedical Engineering Chung Yuan Christian University Taoyuan Taiwan

**Keywords:** cognitive load, heart rate variability, video learning, learning outcome, secondary-task reaction time, virtual reality

## Abstract

**Background:**

Learning through a 360° virtual reality (VR) or 2D video represents an alternative way to learn a complex medical education task. However, there is currently no consensus on how best to assess the effects of different learning materials on cognitive load estimates, heart rate variability (HRV), outcomes, and experience in learning history taking and physical examination (H&P) skills.

**Objective:**

The aim of this study was to investigate how learning materials (ie, VR or 2D video) impact learning outcomes and experience through changes in cognitive load estimates and HRV for learning H&P skills.

**Methods:**

This pilot system–design study included 32 undergraduate medical students at an academic teaching hospital. The students were randomly assigned, with a 1:1 allocation, to a 360° VR video group or a 2D video group, matched by age, sex, and cognitive style. The contents of both videos were different with regard to visual angle and self-determination. Learning outcomes were evaluated using the Milestone reporting form. Subjective and objective cognitive loads were estimated using the Paas Cognitive Load Scale, the National Aeronautics and Space Administration Task Load Index, and secondary-task reaction time. Cardiac autonomic function was assessed using HRV measurements. Learning experience was assessed using the AttrakDiff2 questionnaire and qualitative feedback. Statistical significance was accepted at a two-sided *P* value of <.01.

**Results:**

All 32 participants received the intended intervention. The sample consisted of 20 (63%) males and 12 (38%) females, with a median age of 24 (IQR 23-25) years. The 360° VR video group seemed to have a higher Milestone level than the 2D video group (*P*=.04). The reaction time at the 10th minute in the 360° VR video group was significantly higher than that in the 2D video group (*P*<.001). Multiple logistic regression models of the overall cohort showed that the 360° VR video module was independently and positively associated with a reaction time at the 10th minute of ≥3.6 seconds (exp B=18.8, 95% CI 3.2-110.8; *P*=.001) and a Milestone level of ≥3 (exp B=15.0, 95% CI 2.3-99.6; *P*=.005). However, a reaction time at the 10th minute of ≥3.6 seconds was not related to a Milestone level of ≥3. A low-frequency to high-frequency ratio between the 5th and 10th minute of ≥1.43 seemed to be inversely associated with a hedonic stimulation score of ≥2.0 (exp B=0.14, 95% CI 0.03-0.68; *P*=.015) after adjusting for video module. The main qualitative feedback indicated that the 360° VR video module was fun but caused mild dizziness, whereas the 2D video module was easy to follow but tedious.

**Conclusions:**

Our preliminary results showed that 360° VR video learning may be associated with a better Milestone level than 2D video learning, and that this did not seem to be related to cognitive load estimates or HRV indexes in the novice learners. Of note, an increase in sympathovagal balance may have been associated with a lower hedonic stimulation score, which may have met the learners’ needs and prompted learning through the different video modules.

**Trial Registration:**

ClinicalTrials.gov NCT03501641; https://clinicaltrials.gov/ct2/show/NCT03501641

## Introduction

### Competence-Based Medical Education Needs a Multifaceted Assessment System

From premedical students to practicing physicians, competency-based medical education (CBME) has been applied in the design, implementation, and evaluation of medical education programs according to competency-based outcomes [1]. CBME for undergraduate medical students can help to improve task-specific confidence and test performance after the course has been completed, resulting in better performance and patient care before residency training [2]. CBME has been widely employed to promote greater learner-centeredness and curricular outcomes since 2010 [3]. Furthermore, CBME must use multiple assessment tools that meet minimum requirements for quality. Therefore, a robust and multifaceted assessment system, including quantitative and qualitative methods, is essential for evaluating the progress of learners [4].

### Simulation Provides Situated Learning and Assessment in a Safe Environment

CBME is used to improve graduates’ competency levels, ensuring they are skillful and qualified in all critical areas of their occupation. To this end, CBME emphasizes the use and importance of simulation-based training, which considers patient safety and for which real-life opportunities are limited [5]. This consideration is crucial for medical learners and teachers during the COVID-19 pandemic. History taking and physical examination (H&P) is a principal competency, incorporating knowledge, skills, and behavior to initially approach a patient. Therefore, H&P is a key performance level of otorhinolaryngology–head and neck surgery (ORL-HNS) [6]. To enhance the development of H&P skills, commonly used methods to assess this competency include in-training examinations such as the Milestone [7], Mini Clinical Evaluation Exercise [8], and oral examinations of clinical practice [9]. Recently, simulations such as part-time trainers, integrated simulators, virtual reality (VR), and wearable devices have become increasingly popular for CBME [10]. Simulation-based training, such as VR, has been shown to improve health professionals’ knowledge and skills outcomes and be an integrative step toward supervised clinical practice [11]. In particular, VR allowed students to practice rounding skills, facilitate their education during the COVID-19 pandemic, and supplement their in-person clerkship education [12].

### 360° VR Video Can Provide High Authenticity and Fidelity to Encourage Learners

VR consists of a computer-generated 3D simulation in which the user both explores and manipulates the contents of the environment to learn and assess clinical knowledge and skills [13]. VR provides experiential learning and provides standardized, controlled exposure to situated events, patient-caregiver communication, and teamwork. The use of VR has been shown to be highly acceptable by learners in a wide range of health care settings [14] and to play an essential role in improving performance [15]. As a subtype of image-based VR, 360° VR video represents an immersive 3D medium featuring authenticity and fidelity using a VR head-mounted display. The use of 360° VR video opens up many possibilities in many domains of medical education [16], such as an independent teaching aid or an adjunct to traditional face-to-face teaching [17]. The application of 360° VR video has enhanced the effectiveness of medical education and training, raised the level of diagnosis and treatment, improved the doctor-patient relationship, and boosted medical execution efficiency [18]. Using 360° VR videos to facilitate the acquisition of new clinical skills has been suggested to be a valuable step in developing a clinical teaching curriculum [19].

### Incorporating Instructional Design Practices That Address the Elements of Cognitive Load Theory Is Important to Improve Learning Outcomes

However, transferring practical skills to a clinical setting using a criterion-based training program with VR simulators is difficult [20]. In addition to the poor mechanical performance of the simulated haptic feedback, complex tasks such as H&P and surgical procedures can induce excessive cognitive load during simulation training, which can harm learning, especially for novices [21]. Since the learner’s cognitive capacity during learning is limited, improper instructional design may waste precious cognitive resources, impair the essential processing, and cause generative underutilization during learning [22]. Therefore, cognitive overload negatively impacts learning outcomes in simulation training [23,24]. For example, the increased cognitive load was significantly associated with the declined correct identification of a trained murmur during simulation training [23]. A study of laparoscopic training [24] reported that immersive VR simulation training resulted in the cognitive overload that impeded actual learning and skills acquisition compared with conventional VR simulation training. However, structured and distributed VR simulation practices may induce a lower cognitive load when the learning situation is increased in complexity [21]. Recently, VR-based simulation training under cognitive load control has improved performance under similar conditions to an actual surgical task [25]. Therefore, estimating cognitive load during 360° VR and 2D video learning is essential for practical instruction and reform.

### Estimating Cognitive Load During 360° VR Video Learning Is a Challenge

Estimations of cognitive load and specific load types are still challenging based on current cognitive load theory [26,27]. For example, subjective cognitive load questionnaires, such as the Paas Cognitive Load Scale (Paas-CLS) [28] and the National Aeronautics and Space Administration Task Load Index (NASA-TLX) [29] have been shown to be good tools to measure intrinsic load, but not extraneous and germane loads [30]. However, a lowering of intrinsic cognitive load can reduce total cognitive load, thus releasing working memory capacity [31]. Furthermore, instructional techniques for reducing cognitive load have been shown to improve learning [32]. In addition to estimating cognitive load, the NASA-TLX has been successfully applied to determine user acceptance when evaluating innovative applications [33]. Nevertheless, a negative carryover effect due to failure in a VR simulation training program could affect the subjective cognitive load estimations [34]. Therefore, objective estimates of cognitive load, including secondary-task performance [35], during the training program have been applied in many medical education studies [34-36]. Secondary-task performance is a task that assesses participants’ attention and is limited by the storage capacity of visual short-term memory [37]. Although secondary-task performance is a cognitive function test, it frequently deteriorates from baseline to dual-task among novices and is particularly useful for tracking changes in cognitive load in the early phases of simulation-based skills [35].

### Autonomic Function Can Be Altered During VR Immersion

VR immersion, especially containing stressful content, can evoke acute stress reactions accompanied by autonomic dysfunction [38]. Heart rate variability (HRV) is a sensitive indicator of cardiac autonomic modulation [39] and responds to any psychophysical changes immediately. Several physiological or psychological changes, such as stress [40] and anxiety [41], can change HRV. Reduced HRV indexes might reflect loss of cognitive efficiency [36] and might predict increased cognitive load [42] and poor cognitive performance [43]. Nevertheless, immersive VR using the first-person perspective can induce body ownership illusion in an uncomfortable posture, reduced HRV indexes, and more mistakes in a cognitive task [44]. Despite it being challenging to assess associations between HRV and subjective cognitive load due to sizeable interindividual variability [45], measuring HRV during a learning task may help evaluate whether learning materials impact learning outcomes through cardiac changes autonomic function.

### Aims and Hypotheses of the Study

This study aimed (1) to evaluate differences between two learning materials (ie, 360° VR video and 2D video) in the subjective and objective cognitive loads, autonomic function, outcome, and learning experience while students were learning H&P skills and (2) to study how learning materials (ie, 360° VR or 2D video) impact learning outcomes through the changes in cognitive load and HRV. The initial hypotheses were that (1) subjective and objective cognitive loads and autonomic function would be changed while using different learning materials and (2) learners with higher learning outcomes and experience would have different cognitive load and autonomic function during the learning tasks than those with lower learning outcomes and experience.

Therefore, we used cognitive questionnaires immediately after completing a video learning module to assess subjective cognitive load. We also used secondary-task reaction time and HRV to objectively estimate the dynamic changes of cognitive load and cardiac autonomic function during a video module for providing ongoing feedback to improve video learning. Subsequently, we used the Milestone report form to evaluate the real-patient H&P. The Milestones have been designed according to the grounding principles of CBME and have become a significant formative component of the current accreditation model of graduate medical education [46]. Although the Milestones were not designed to assess undergraduate medical students, we considered that some descriptors and targets of the Milestones were suitable for evaluating learner performance of H&P as an undergraduate medical student moves from entry into clerkship through graduation. Furthermore, we applied the AttrakDiff2 questionnaire to assess the acceptance of technical innovations [47]. The AttrakDiff2 questionnaire has been used to investigate learners’ experience of many educational innovations, such as mobile e-learning [48] and augmented reality [49]. Finally, we used anonymous qualitative feedback to determine the learning experience. We considered that the learning outcomes and the learning experience could meet learners’ needs and prompt their use of learning through different video modules.

## Methods

### Study Design

**Figure 1:**
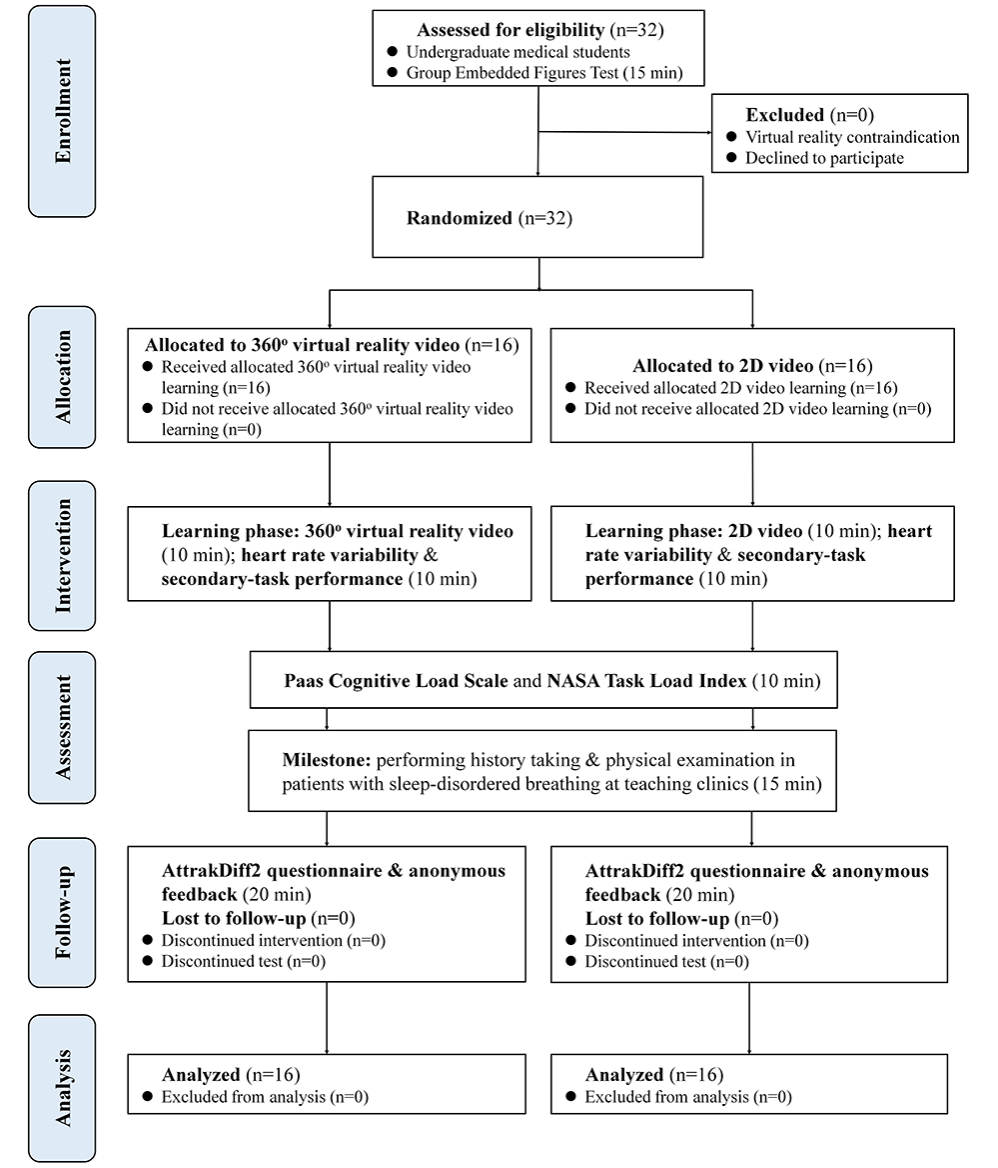
The CONSORT flow diagram. NASA: National Aeronautics and Space Administration.

We conducted a prospective, randomized controlled, pilot system–design study from June 1 to October 30, 2018, at an academic teaching hospital (Department of ORL-HNS, Linkou Chang Gung Memorial Hospital, Taoyuan, Taiwan). The Institutional Review Board of the Chang Gung Medical Foundation approved this study (No. 201601821B0), and we conducted all procedures in compliance with the Declaration of Helsinki 1975. We informed the participants about the aims of the study and then obtained written informed consent from them. We registered the entire study proposal at ClinicalTrials.gov (NCT03501641). Figure 1 shows the study flowchart following the CONSORT 2010 guidelines [50].

### Setting

#### Overview

We used analysis, design, development, implementation, and evaluation models [51] to design an effective instruction module for H&P, including essential knowledge and competence according to the guidelines of the American Board of Otolaryngology [7]. We used different working samples [52], including instructions for how to formulate problems, solution steps, and final solutions, to demonstrate, step by step, how to perform an H&P task in an outpatient setting. We also used self-explanation prompts [53] to encourage the learner to recognize links between the knowledge and skills they learned. We recorded a 10-minute 360° video (4K resolution, 30 frames/s) with in-camera stitching, capturing 360° audio, and spherical stabilization using a 360° camera (Garmin VIRB 360; Garmin Ltd). We constructed the contents and scenario of this video according to a real clinical setting. The first portion of the video demonstrated skills of history taking under normal conditions, and the second portion demonstrated skills of how to quickly perform a physical examination (Table 1). Subsequently, we produced two videos with different visual angles (ie, 360° and 120°) using PowerDirector software (version 16; CyberLink Corp). Two senior investigators evaluated the videos and validated the learning materials. We then developed courseware with the same user interface for the 360° VR and 2D videos using Unity Editor (version 2017.3.1; Unity Technologies).

#### The 360° VR Video Module

**Figure 2:**
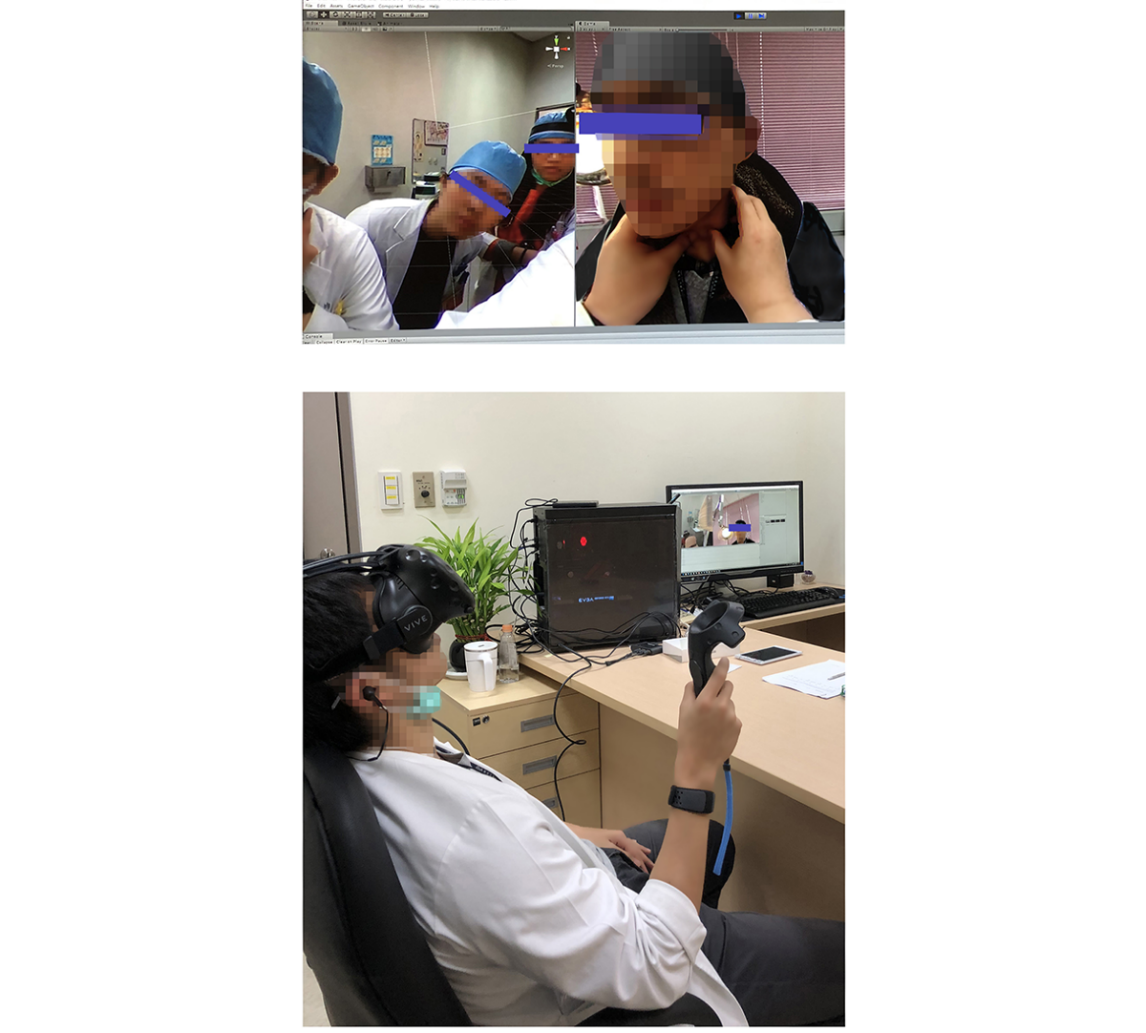
Example of 360° virtual reality video learning. Screenshot of the 360° virtual reality video (upper) demonstrating the learners watching a highly immersive 360° video. They arbitrarily changed their field of view to watch the skills of history taking and physical examination from a first-person perspective (lower).

This module was developed to arbitrarily review the immersive 3D 360° VR video through a head-mounted display (Figure 2). The users were immersed in the 360° experience to learn the H&P skills.

#### The 2D Video Module

**Figure 3:**
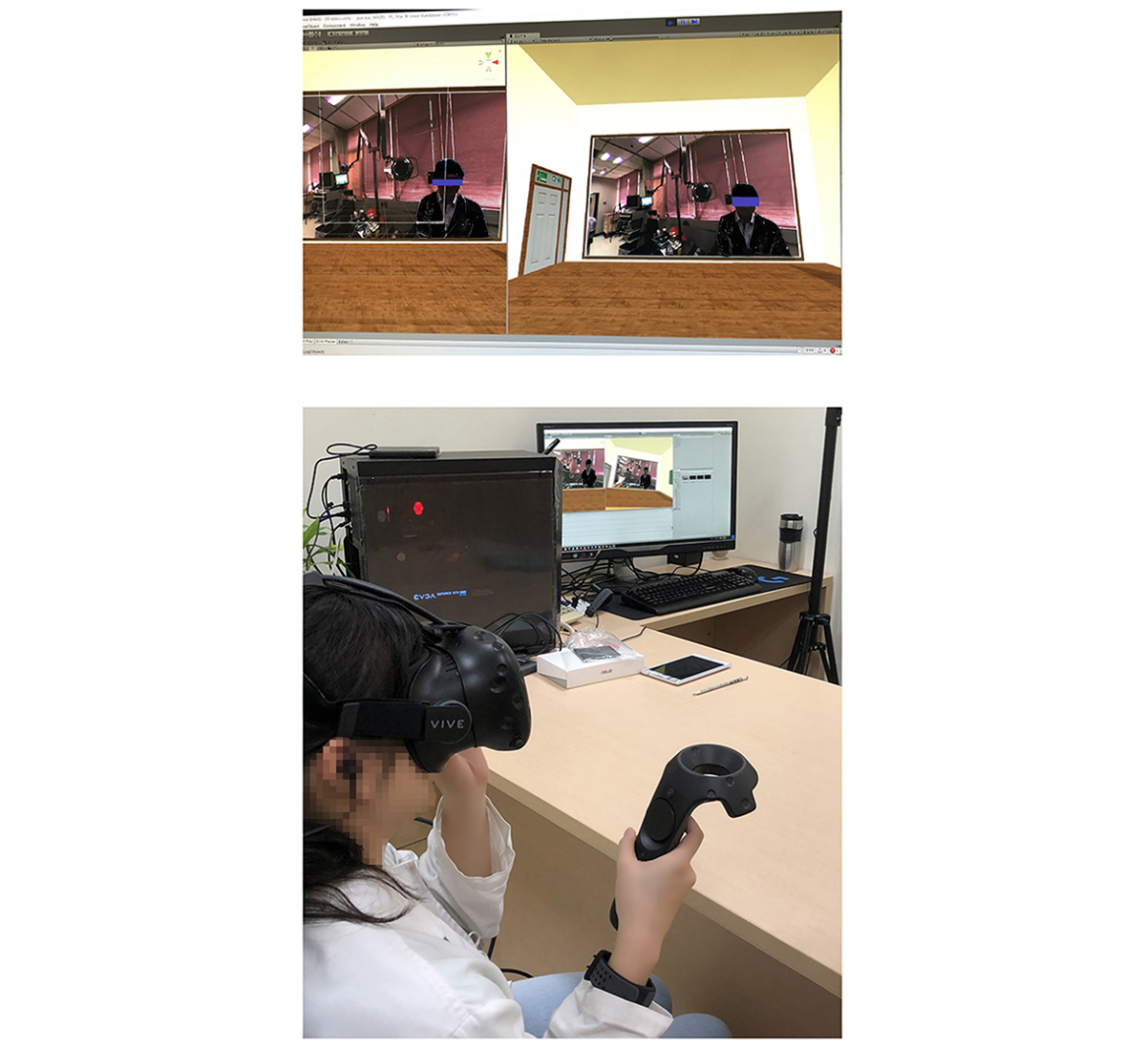
Example of 2D video learning. Screenshot of the 2D video (upper) showing the learners watching a 2D video in a theater environment. They watched the skills of history taking and physical examination in a fixed focus of view from a third-person perspective (lower).

The 2D video was played in a fixed 120° focused field of view through the same head-mounted display (Figure 3). The users reviewed the instructional video as in a theater environment.

**Table 1:** Summary of the models of 360° virtual reality video and 2D video.

Feature	360° virtual reality video module	2D video module
Head-mounted display	Yes	Yes
Part I: history taking	Statically filmed	Statically filmed
Part II: physical examination	Dynamically filmed	Dynamically filmed
Visual angle	360°	120°
Immersion	Yes	No
Perspective	First person	Third person

### Selection of Participants

A convenience sampling approach was used, and 32 consecutive volunteers were recruited during the study period. The inclusion criteria were as follows: (1) aged >20 years and (2) novices in ORL-HNS (ie, undergraduate medical students). The exclusion criteria were as follows: (1) contraindications for using VR, such as pregnancy, hypertension, motion sickness, inner ear infections, claustrophobia, recent surgery, pre-existing binocular vision abnormalities, heart disorders, or epilepsy, and (2) declining to participate. All of the volunteers had at least a basic level of computer literacy and could use VR headsets and controllers after instruction. We used the 25-item Group Embedded Figures Test (GEFT) (score range 0-18) to assess the participants’ cognitive style [54]. The GEFT has been shown to have high reliability in medical education [55], and we have previously validated its effectiveness of classifying learning preference in millennial undergraduate medical students [56]. Field-independent learners prefer and have better performance in computer-assisted learning. We stratified the students into two subgroups: “field-dependent” (GEFT score ≤12) and “field-independent” (GEFT score >12) [57].

### Randomization and Blinding

The participants were blinded to the purpose of the study during recruitment to minimize preparation bias. After the participants had provided consent and completed the GEFT, we randomly assigned them, with a 1:1 allocation, to the 360° VR video group and the 2D video group, matched by age, sex, and cognitive style (Figure 1). The Random Number Generators tool in SPSS software (version 24; IBM Corp) was used to create a list of random numbers for allocating the students, who were stratified by center with a 1:1 allocation using a fixed block size of 8 in both parallel subgroups. We concealed the allocation sequence before implementing the video module, and the module adhered to our computer-generated randomization protocol.

### Intervention

After randomization, the participants reviewed their allocated video through a head-mounted display in the same space for 10 minutes. To reduce the effect of the head-mounted display on learning experience, both groups used the same VR device (VIVE VR headset; HTC Corp). We explained the functionality of the VR device to the participants before the intervention. In the 360° VR video group, the learners arbitrarily reviewed the instructor’s demonstrations and responses from standard patients and other medical staff from a first-person perspective in an immersive 360° environment (Figure 2). In the 2D video group, the learners simply watched the instructor’s demonstrations from a third-person perspective in a theater environment (Figure 3). During the learning course, the participants watched the movie by themselves at a time of their choosing.

### Methods of Measurement

#### Overview

We used five different face-to-face assessments, including the Paas-CLS and the NASA-TLX for subjectively estimating the cognitive load, the Milestone for assessing the learning outcomes, and the AttrakDiff2 questionnaire and anonymous qualitative feedback for determining the learning experience. There was one objective estimate of cognitive load using the secondary-task performance and one measurement of cardiac autonomic function using HRV monitoring.

#### Paas Cognitive Load Scale

We used the Paas-CLS [28] to estimate the total cognitive load of the learning task immediately after the intervention. The Paas-CLS questionnaire is a single-item measure to rate the perceived intensity of mental effort along a 9-point scale, ranging from 1 (very, very low mental effort) to 9 (very, very high mental effort). The Paas-CLS questionnaire has good reliability (Cronbach α=.82-.90) in instructional research [58].

#### NASA Task Load Index

The NASA-TLX questionnaire is a subjective assessment of cognitive load [29]. This instrument consists of six subscales: mental demand, physical demand, temporal demand, performance, effort, and frustration. Participants rate the level of each dimension by making a mark on a visual analog scale (range 0-20) immediately after the intervention. The NASA-TLX questionnaire has good reliability (Cronbach α≥.80) to assess cognitive load [59].

#### Secondary-Task Performance

Secondary-task performance has been shown to be sensitive in estimating intrinsic cognitive load among novices engaged in simulation-based learning [35]. As the primary task in this study, the participants reviewed the video for 10 minutes. To ensure the use of similar perceptual-cognitive resources for the primary task [60], the participants were asked to respond to a visual cue by pressing a button on a controller as soon as possible. The visual cue appeared in their field of view and lasted for 10 seconds. For exploring the importance of monitoring secondary-task reaction time, we measured the reaction time, deﬁned as the time from visual cue presentation to when the button was pressed, at 0 minutes, 5 minutes, and 10 minutes during the reviewing period. When the participants missed a response, we considered the reaction time to be “11 seconds” to avoid gaps in the performance results and inaccuracies in the estimates of the reaction time.

#### Heart Rate Variability

HRV has been shown to be an objective estimation of learners’ cognitive load in a learning environment [61,62]. For controlling preinterventional stress, the participants sat quietly for 20 minutes. We recorded 5-minute electrocardiogram signals of a single lead (lead I) using a Holter-like NeXus-4 amplifier and recording system (Mind Media BV) when the participant wore a head-mounted display and breathed normally as baseline data. During the 10-minute intervention, we recorded heart rates simultaneously and continuously. Electrocardiogram data were acquired with a sample rate of 1024 Hz, and the raw data were saved. The power spectrum was quantified by a fast Fourier transform [39]. In our preliminary results, the energy-frequency-time distributions of the electrocardiogram data were nonlinear and nonstationary. Therefore, we used the empirical mode decomposition (EMD) method to decompose complicated data into a finite number of intrinsic mode functions that admitted well-behaved Hilbert transforms [63]. The EMD method is an adaptive preprocessing technique for overcoming the limitations of HRV spectral analysis when assessing nonlinear and nonstationary system data [64]. We performed HRV analysis using custom-developed MATLAB (version 7; The MathWorks, Inc) codes that allowed us to determine HRV parameters from sequences of 5-minute consecutive epochs of electrocardiogram signals. The requirements for the quality of HRV analysis were based on the European Society of Cardiology and the North American Society of Pacing and Electrophysiology guidelines [65]. For the analysis, sequences of R wave to R wave (RR) intervals were selected without artifacts, ventricular excitations, or supraventricular excitations [66]. We then calculated the RR interval, the SD of normal-to-normal RR intervals (SDNN), and the root mean square of successive heartbeat interval difference (RMSSD) using time-domain analysis. HRV in the frequency domain was described using spectral power values in the low-frequency (LF) band (0.04-0.15 Hz), the high-frequency (HF) band (0.15-0.40 Hz), and the LF/HF ratio. To reflect the different instructional content, we analyzed three time intervals: baseline, 0 to 5 minutes, and 5 to 10 minutes. These HRV indexes were chosen because low SDNN was associated with higher intrinsic and germane cognitive loads [67] and poor performance [68], low RMSSD was related to higher intrinsic cognitive load [67] and worse performance on executive tasks [69], and low LF/HF ratio was associated with high cognitive load [70]. The HRV variables were measured before and after the intervention.

#### Milestones

The Milestones have been used to assess the development of resident physicians in key dimensions of the elements of physician competency in otolaryngology since 2004 [7]. In this study, we prospectively recruited participants to perform H&P in real patients with sleep-disordered breathing (SDB) at our teaching clinics. For evaluating learner performance of H&P as an undergraduate medical student moves from entry into clerkship through graduation, we selected the level that best described that learner’s performance with the Milestone for care for patients with SDB [6]. We used a brief 5-level Milestone report form to rate learner performance of H&P, including (1) obtaining general H&P, (2) recognizing signs and symptoms of SDB and the differences between children and adults, (3) performing detailed examinations with evaluations of upper airway anatomy, (4) interpreting the examinations and advanced diagnostic testing, and (5) teaching-focused H&P.

#### AttrakDiff2 Questionnaire

The AttrakDiff2 questionnaire was developed to reliably evaluate the acceptance of technical innovations [47]. It assesses qualities of pragmatism, hedonic stimulation, hedonic identification, and attractiveness using 28 questions. The participants were asked to respond to each question by making a mark on a 7-point Likert-like scale, ranging from –3 to 3, with a semantic differential design. Subsequently, the mean value of each quality created a scale value for pragmatism, hedonic stimulation, hedonic identification, and attractiveness. The AttrakDiff2 questionnaire has been applied to evaluate learners’ experience [48,71].

### Anonymous Qualitative Feedback

Each participant in this study provided anonymous feedback about their experience of the module used.

### Outcome Measures

The primary outcome measure of this study was the Milestone level after completing the video learning module. The secondary outcomes were the AttrakDiff2 questionnaire scales.

### Sample Size

The sample size was estimated using primary outcome effects (the Milestone) based on a priori study (360° VR video: mean 3.1, SD 0.7; 2D video: mean 2.3, SD 0.6). A two-tailed Wilcoxon-Mann-Whitney *U* test was used to calculate a sample size of 16 in each group (normal parent distribution; calculated effect size, 1.23; type I error, 0.01; power, 70%). For a block size of 8, we decided to enroll a total of 32 students to show the difference in the Milestone level.

### Statistical Analysis

The D’Agostino-Pearson omnibus normality test showed that most of the continuous variables were nonnormally distributed, and they were presented as median and IQR. Differences between groups were analyzed using the Wilcoxon signed-rank test, the Mann-Whitney *U* test, or the Fisher exact test as appropriate. Effect sizes were calculated using the Hodges-Lehmann method for the Mann-Whiney *U* test and the Wilcoxon signed-rank test, or odds ratios for the Fisher exact test. The Spearman correlation test was used to analyze relationships between variables of interest. The Bonferroni correction was used to adjust *P* values because of the increased risk of a type I error when conducting multiple statistical tests at the same time [72]. Continuous variables were dichotomized using the median split. Variables of interest were analyzed for multivariate logistic regression models. All *P* values were two‐sided, and statistical significance was accepted at *P*<.01. Statistical analyses were performed using G*Power software (version 3.1.9.2; Heinrich-Heine-Universität Düsseldorf), Prism for Windows (version 7.0; GraphPad Software Inc), and SPSS Statistics for Windows (version 25; IBM Corp).

## Results

### Study Participants

A total of 32 volunteers were recruited, of which 20 (63%) were males and 12 (38%) were females. The median age was 24 (IQR 23-25) years. There were 3 (9%) field-dependent and 29 (91%) field-independent participants. Table 2 summarizes the variables of interest for the overall study cohort. As expected, there were no significant differences in age, sex, or cognitive style between the 360° VR video and 2D video groups at baseline. After randomization, all participants received the intended intervention. There were no protocol deviations in this study.

**Table 2:** Demographics and cognitive style.

Variables		Overall (N=32)	360° virtual reality video group (n=16)	2D video group (n=16)	Effect size^a^ (95% CI)	*P* value^b^
**Demographics**						
	Age (years), median (IQR)	24 (23-25)	24 (23-25)	24 (23-25)	0 (–1 to 0)	.29
	Sex (male), n (%)	20 (63)	10 (63)	10 (63)	1.0 (0.2 to 4.2)	>.99
Cognitive style: field-dependent, n (%)		3 (9)	1 (6)	2 (13)	2.1 (0.2 to 26.3)	>.99

^a^Effect sizes were calculated using the Hodges-Lehmann method for the Mann-Whiney *U* test and Wilcoxon signed-rank test, or odds ratios for the Fisher exact test.

^b^*P* values were calculated based on the Mann-Whiney *U* test for continuous variables (two-tailed) or the Fisher exact test for categorical variables (two-tailed).

### Estimates of Subjective and Objective Cognitive Load

#### Paas Cognitive Load Scale

The Paas-CLS showed that the participants had a significantly higher total cognitive load than the reference value of “5” in the overall cohort (*P*=.001) after 10 minutes of video instruction (Table 3). Furthermore, the Paas-CLS score of the 360° VR video group was comparable to that of the 2D video group.

#### NASA Task Load Index

**Figure 4:**
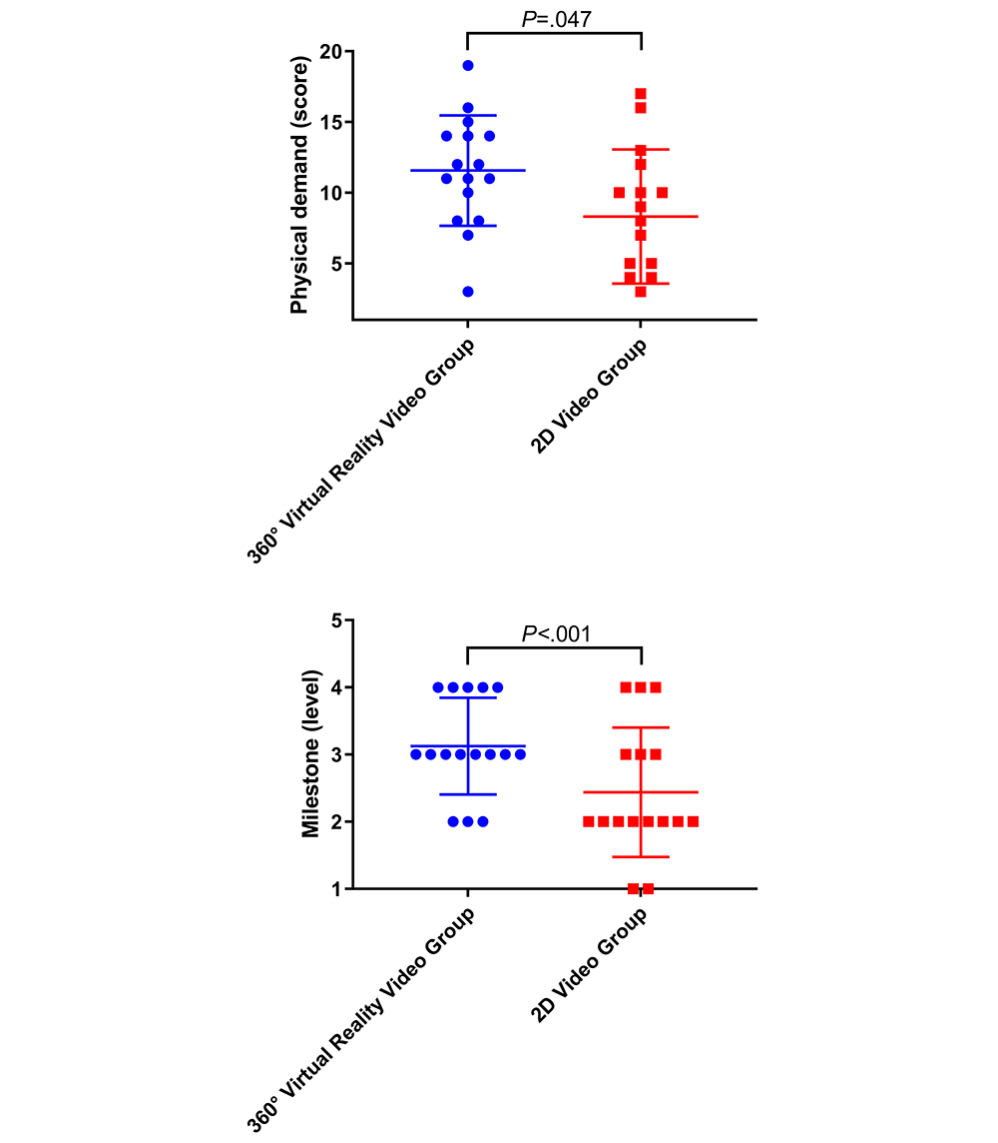
Comparisons of the physical demands and Milestone levels between the 360° virtual reality video group and the 2D video group. The 360° virtual reality video group had a higher physical demand score (upper) and Milestone level (lower) than the 2D video group. However, these differences did not reach statistical significance. *P* values were calculated using the Mann-Whiney *U* test (two-tailed).

The overall cohort had a significantly higher score for mental demand (corrected *P*<.001) than the reference value of “10” before and after the Bonferroni correction (Table 3). The physical demand score of the 360° VR video group was higher than that of the 2D video group; however, this difference did not reach statistical significance after the Bonferroni correction (Figure 4, upper).

**Table 3:** Subjective measures of cognitive load.

Variables		Overall (N=32), median (IQR)	360° virtual reality video group (n=16), median (IQR)	2D video group (n=16), median (IQR)	Effect size^a^ (95% CI)	*P* value^b^
Subjective measurement: Paas Cognitive Load Scale		6 (5-7)^c^	6 (5-7)	5 (5-7)	0 (–1 to 1)	.78
**National Aeronautics and Space Administration Task Load Index**						
	Mental demand	14 (11-15)^c^	14 (12-15)	12 (10-16)	1 (–2 to 3)	.45
	Physical demand	10 (7-14)	12 (9-14)	9 (4-12)	4 (0 to 7)	.047
	Temporal demand	10 (7-11)	10 (8-13)	9 (6-10)	1 (–1 to 4)	.18
	Performance	12 (6-15)	11 (6-15)	13 (6-15)	0 (–4 to 4)	.93
	Effort	12 (10-15)	13 (11-15)	12 (7-15)	1 (–2 to 5)	.45
	Frustration	8 (4-13)	10 (5-13)	7 (3-13)	2 (–2 to 6)	.29

^a^Effect sizes were calculated using the Hodges-Lehmann method for the Mann-Whiney *U* test and the Wilcoxon signed-rank test, or odds ratios for the Fisher exact test.

^b^*P* values were calculated based on the Mann-Whiney *U* test (two-tailed).

^c^*P*<.01, compared with a reference value (score of “5” for the Paas Cognitive Load Scale or score of “10” for the National Aeronautics and Space Administration Task Load Index subscale) and based on the Wilcoxon signed-rank test (two-tailed). *P* values were significant after the Bonferroni correction.

#### Secondary-Task Performance

**Figure 5:**
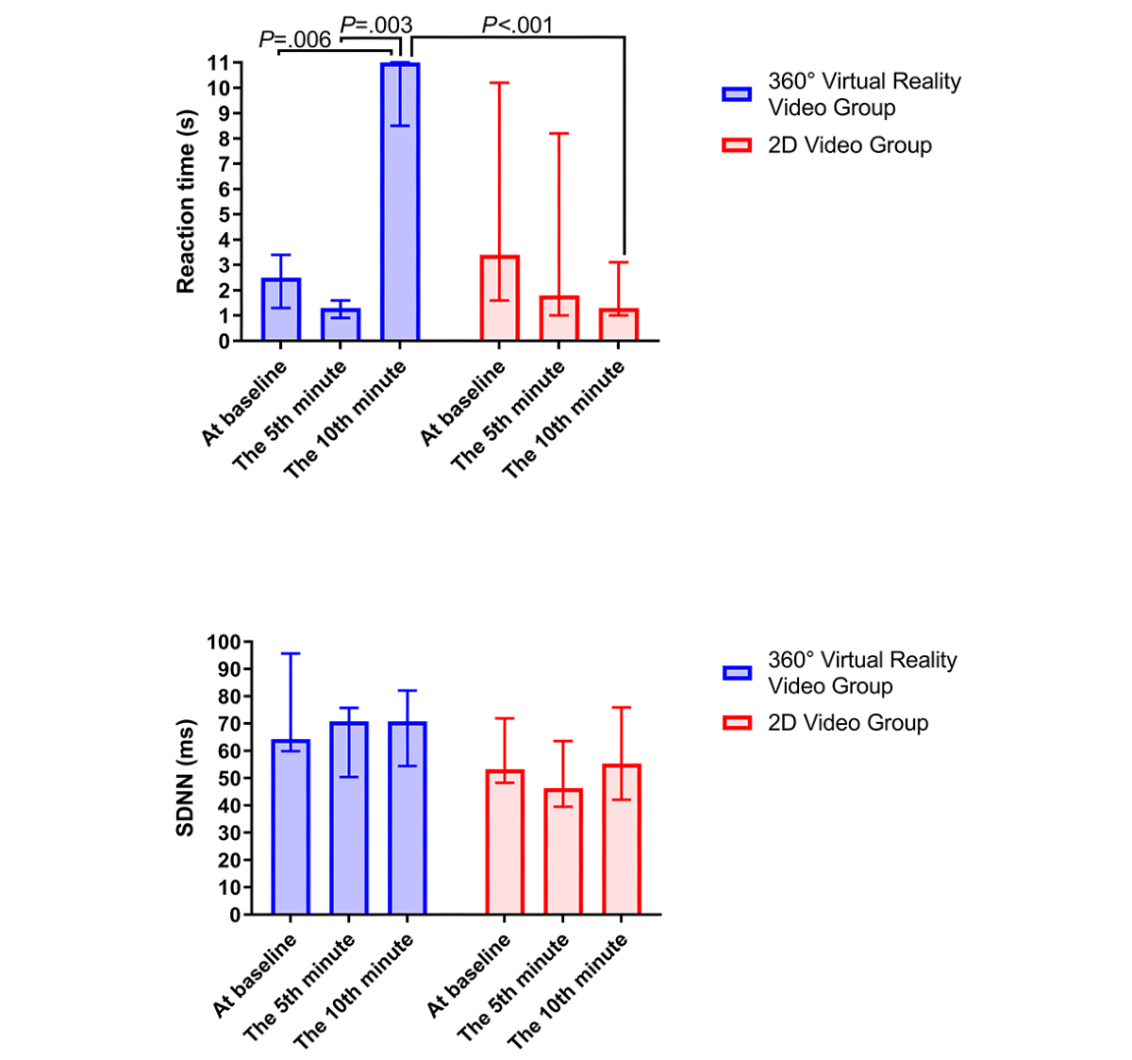
Comparisons of the secondary-task reaction time and SD of normal-to-normal RR intervals (SDNN) between the 360° virtual reality video group and the 2D video group. Notably, the reaction time in the 360° virtual reality video group was significantly higher at the 10th minute compared to the reaction times at baseline and at the 5th minute, whereas the reaction time in the 2D video group was lower at the 10th minute compared to that at baseline. The 360° virtual reality video group had a significantly higher reaction time at the 10th minute (upper) than the 2D video group (upper). The SDNN at 5 to 10 minutes in the 360° virtual reality video group was lower than the SDNN at baseline (lower). *P* values were calculated using the Wilcoxon signed-rank test or Mann-Whitney *U* test as appropriate after the Bonferroni correction.

In the overall cohort (Table 4), the reaction time at the 10th minute was significantly higher than the reaction time at the 5th minute (corrected *P*=.003) but comparable to the reaction time at baseline. In contrast, the reaction time at the 5th minute was equal to the reaction time at baseline after the Bonferroni correction. In the 360° VR video group, the reaction time at the 10th minute was significantly higher than the reaction time at the 5th minute (corrected *P*=.003) and the reaction time at baseline (corrected *P*=.006). Still, the reaction time at the 5th minute and the reaction time at baseline were comparable (Figure 5, upper). In the 2D video group, differences in the reaction time across various time points were not statistically significant. Furthermore, the reaction time at the 10th minute of the 360° VR video group was significantly higher than that of the 2D video group (corrected *P*<.001), even though differences in the reaction time at the 5th minute and reaction time at baseline were not statistically significant.

### Heart Rate Variability

Differences across the RR at 5 to 10 minutes, RR at 0 to 5 minutes, and RR at baseline were not statistically significant in the overall cohort, the 360° VR video group, and the 2D video group, respectively. Furthermore, the RR at 5 to 10 minutes, RR at 0 to 5 minutes, and RR at baseline were comparable between both groups (Table 4). Differences in the SDNN index were not statistically significant for either intragroup comparisons or intergroup comparisons. Differences in the RMSSD index were not statistically significant for either intragroup comparisons or intergroup comparisons. Differences in the LF/HF index were not statistically significant for either intragroup comparisons or intergroup comparisons.

**Table 4:** Objective estimates of cognitive load.

Variables		Overall (N=32), median (IQR)	360° virtual reality video group (n=16), median (IQR)	2D video group (n=16), median (IQR)	Effect size^a^ (95% CI)	*P* value^b^
**Secondary-task performance**						
	Reaction time–baseline (s)	2.6 (1.4-7.4)	2.5 (1.3-3.4)^c,d^	3.4 (1.6-10.2)	–0.6 (–5.0 to 0.7)	.34
	Reaction time–5th min (s)	1.6 (0.9-3.6)^d,^^e^	1.3 (0.9-1.6)^d,e^	1.8 (1.0-8.2)	–0.4 (–2.0 to 0.13)	.18
	Reaction time–10th min (s)	3.6 (1.3-11.0)^d,e^	11.0 (7.5-11.0)^c^^,^^d,^^e^	1.3 (1.0-3.1)^d^	8.3 (3.6 to 9.8)^d^	<.001
**Heart rate variability**						
	RR–baseline (ms)	810 (741-918)	825 (742-937)	802 (721-891)	25 (–75 to 122)	.62
	RR–0 to 5 min (ms)	810 (730-908)	833 (762-925)	772 (724-884)	45 (–42 to 143)	.31
	RR–5 to 10 min (ms)	779 (722-889)	794 (736-911)	779 (709-866)	20 (–64 to 117)	.75
	SDNN–baseline (ms)	69.9 (48.7-135.1)	88.3 (65.7-139.0)	57.3 (42.7-135.6)	25.3 (–6.1 to 65.6)	.10
	SDNN–0 to 5 min (ms)	59.8 (45.5-151.3)	72.0 (58.6-184.4)	51.7 (39.2-107.7)	20.1 (–6.4 to 95.3)	.11
	SDNN–5 to 10 min (ms)	69.8 (42.3-121.9)	71.2 (51.7-128.2)	58.9 (35.8-91.7)	16.3 (–20.9 to 52.2)	.27
	RMSSD–baseline (ms)	60.7 (31.7-179.4)	106.3 (39.7-189.1)	34.3 (28.6-175.1)	29.8 (–6.7 to 107.2)	.13
	RMSSD–0 to 5 min (ms)	63.0 (30.4-203.8)	78.0 (34.5-252.4)	37.4 (23.8-133.7)	24.8 (–12.9 to 129.0)	.29
	RMSSD–5 to 10 min (ms)	54.9 (27.8-145.0)	72.1 (31.9-158.5)	39.4 (27.5-96.8)	8.7 (–17.9 to 71.5)	.51
	LF/HF ratio–baseline	1.02 (0.78-1.96)	0.86 (0.77-1.96)	1.21 (0.79-1.96)	–0.13 (–0.63 to 0.39)	.59
	LF/HF ratio–0 to 5 min	1.19 (0.72-2.83)	0.93 (0.67-3.35)	1.59 (0.77-2.34)	–0.11 (–1.09 to 1.11)	.91
	LF/HF ratio–5 to 10 min	1.43 (0.86-2.80)	1.43 (0.74-2.89)	1.43 (0.89-2.86)	–0.04 (–0.84 to 0.79)	.81

^a^Effect sizes were calculated using the Hodges-Lehmann method for the Mann-Whiney *U* test.

^b^*P* values were calculated based on the Mann-Whiney *U* test (continuous variables).

^c^*P*<.01, compared with the baseline value, using the Wilcoxon signed-rank test (two-tailed).

^d^*P* values were significant after the Bonferroni correction.

^e^*P*<.01, compared with the 5th-minute value of reaction time or the 0-to-5–minute values of R wave to R wave (RR), standard deviation of normal-to-normal RR intervals (SDNN), root mean square of successive heartbeat interval difference (RMSSD), low frequency (LF), high frequency (HF), or LF/HF ratio, using the Wilcoxon signed-rank test (two-tailed).

### Primary Outcome: Milestone Level

Overall, the participants had a significantly higher Milestone level (median 3, IQR 2-4) than the reference value of “1” (*P*<.001) after 10 minutes of video instruction. Although the Milestone level of the 360° VR video group (median 3, IQR 3-4) was higher than that of the 2D video group (median 2, IQR 2-3), the difference did not reach statistical significance (effect size=1, 95% CI 0-1; *P*=.02) (Figure 4, lower).

### Secondary Outcomes: AttrakDiff2 Questionnaire Scales

The overall cohort and the 360° VR and 2D video groups had significantly positive learning experiences in terms of pragmatic quality, hedonic stimulation, hedonic identification, and attractiveness, compared with the reference value of “0” with all corrected *P*<.01, after 10 minutes of video instruction (Table 5). There were no statistically significant differences in pragmatic quality, hedonic stimulation, hedonic identification, or attractiveness between the two groups.

**Table 5:** Acceptance of technical innovations assessed by the AttrakDiff2 questionnaire.

Variables	Overall (N=32), median (IQR)^a^	360° virtual reality video group (n=16), median (IQR)^a^	2D video group (n=16), median (IQR)^a^	Effect size^b^ (95% CI)	*P* value^c^
Pragmatic quality	1.9 (1.2-2.3)	2.0 (1.2-2.4)	1.8 (1.2-2.3)	0.1 (–0.4 to 0.7)	.59
Hedonic stimulation	2.0 (1.0-2.5)	2.1 (1.2-2.6)	2.0 (0.9-2.4)	0.1 (–0.4 to 0.7)	.42
Hedonic identification	1.9 (1.2-2.4)	1.8 (1.5-2.4)	2.1 (1.5-2.9)	0.1 (–0.6 to 0.9)	.90
Attractiveness	1.3 (0.9-2.1)	1.2 (1.1-1.9)	1.6 (0.6-2.3)	–0.1 (–0.9 to 0.7)	.84

^a^*P*<.01 for all variables, compared with a reference value of “0” for the AttrakDiff2 questionnaire using the Wilcoxon signed-rank test (two-tailed). *P* values were significant after the Bonferroni correction.

^b^Effect sizes were calculated using the Hodges-Lehmann method for the Mann-Whiney *U* test.

^c^*P* values were calculated based on the Mann-Whiney *U* test for continuous variables (two-tailed).

### Correlations Between the Learning Modules, Cognitive Load Estimates, Learning Outcomes, and Technical Acceptance

**Figure 6:**
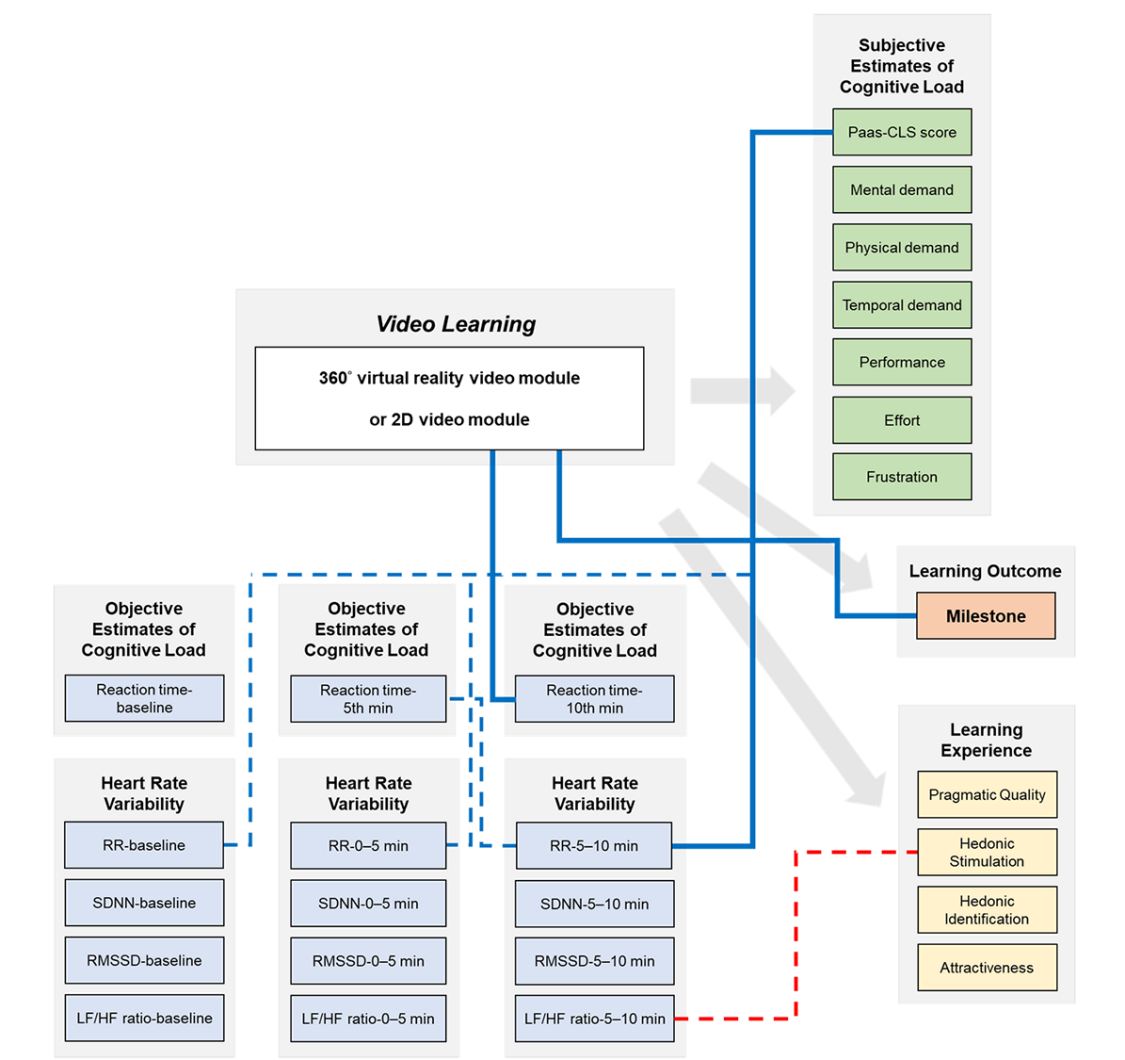
Associations between the video learning modules, cognitive load measures, heart rate variability, learning outcome, and learning experience. Solid blue lines indicate an independent positive association between two variables after adjustment for video module using multivariate logistic regression models. In contrast, dashed blue lines indicate a positive association, and a dashed red line shows an inverse association without statistical significance after adjustment for the video module. HF: high frequency; LF: low frequency; Paas-CLS: Paas Cognitive Load Scale; RMSSD: root mean square of successive heartbeat interval difference; RR: R wave to R wave; SDNN: SD of normal-to-normal RR intervals.

To investigate how learning materials impact learning outcomes through the changes in cognitive load and HRV, we dichotomized the Milestone level, AttrakDiff2 questionnaire scales, cognitive load variables, and HRV indexes. Figure 6 demonstrates the associations between variables of interest.

### Qualitative Evaluation

#### Benefits

The 360° VR video group reported that the 360° VR video module was “fun to learn” (3/16, 19%) and “good for physical examinations” (1/16, 6%). Qualitative feedback from the 2D video group emphasized that they found the 2D video module “easy to follow” (2/16, 13%) and “highly efficient” (1/16, 6%).

#### Faults

Out of 16 participants in the 360° VR video group, 3 (19%) reported that it caused “mild VR dizziness.” Notably, the 2D video learners said that the module was “tedious” (3/16, 19%) and that there was “no interaction” (1/16, 6%).

## Discussion

### Principal Findings

This study’s main findings highlighted the complexities of cognitive load estimations and HRV indexes inherent in developing and evaluating 360° VR and 2D video learning. Our results showed that video learning resulted in a higher total cognitive load and mental demand and prolonged the reaction time at the 10th minute; however, video learning also enhanced a Milestone level of H&P skills with positive learning experiences. Using the same head-mounted display, both the 360° VR and 2D videos were efficient learning methods with positive learning experiences for novice learners. Notably, the 360° VR video learners demonstrated a higher Milestone level of H&P in actual patients with SDB than the 2D video learners. Although both video modules produced comparable total cognitive load and subscales, the 360° VR video learners had a more prolonged reaction time at the 10th minute than the 2D video learners.

Interestingly, in the second portion of the video, the 360° VR video learners had a longer reaction time than the baseline data. These findings suggested that the immersive 360° VR video could reduce secondary-task performance without increasing subjective cognitive loads. Furthermore, the elevated LF/HF ratio at 0 to 5 minutes correlated with a reduced hedonic stimulation scale. Combined with qualitative feedback, we found that the students seemed to consume more cognitive resources to fit the immersive 360° VR video than the 2D video learning. This preliminary study suggests that the immersive 360° VR video might lead to a better Milestone level than the 2D video, which seemed to be unrelated to cognitive load estimates and HRV indexes in novice learners.

### Limitations

This study used quantitative and qualitative measures and methods to evaluate different video learning materials and showed its potential clinical applicability for learning H&P skills. However, several limitations should be addressed. First, this study needs external validation due to the small sample size. Although H&P is one of the essential training topics for undergraduate medical students, our outcomes may not be generalizable to junior residents who already have basic knowledge, skills, and attitudes. Second, assessments of the learner’s performance in the workplace, such as the Mini Clinical Evaluation Exercise, were not included in this study. Third, there were several significant associations between variables of interest in this study. However, the potential relationships between video module, cognitive load, and HRV might be underestimated using such stringent criteria for multiple tests [73]. Accordingly, further studies with a larger sample size with the extended competence spectrum and learning outcome assessments are warranted to confirm our results and inferences.

### Comparison With Prior Work

Our results indicate that both 360° VR and 2D video learning modules can increase H&P competency. Traditionally, students learn clinical skills, such as H&P, from clinical teachers. Instructional videos offer an excellent pedagogical approach to enhance medical students’ clinical competencies and self-confidence levels [74,75]. Moreover, using videos as a delivery format can improve the effects and attractiveness of text-based e-learning [76]. Therefore, clinical teachers can use videos as learning resources for students’ independent learning [77]. Recently, many universities and teaching institutes have promoted the use of instructional videos as a resource for self-directed education and student-centered programs, especially during the COVID-19 pandemic.

Immersive 360° VR videos allow for a virtual experience where learners can improve their competencies by experiencing all of the sights and sounds of teaching clinics and responses from the patients, families, and medical staff from a first-person perspective. When working memory and information processing capacities are limited, 360° VR video learning can trigger a higher cognitive load or stress than conventional 2D video learning. In this study, 360° VR video learning might reduce participants’ attention toward the secondary task without changing subjective cognitive load estimates and HRV indexes regardless of the learning outcome. Therefore, the quality of 360° VR video learning modules still needs to be improved concerning reducing objective cognitive load.

Approximately 25% of our volunteers reported high vibration, out-of-focus blur, eye fatigue, and motion sickness in the preparation phase of the 360° VR video production. A literature review revealed that immersive 360° VR video might induce 3D visual fatigue [78] and visually induced motion sickness [79,80] and may interrupt autonomic balance [81]. Therefore, we used spherical stabilization to smooth quick movements and vibrations and dynamic depth of field to reduce visual discomfort [82]. In addition, we chose a 10-minute instructional course to reduce these known side effects. Although levels of cardiac autonomic balance, as reflected by the LF/HF ratio, were similar during the 360° VR video learning and equivalent to those of the 2D video learning, our 360° VR video learners still experienced a more prolonged secondary-task reaction time, mainly in the second portion of the video.

In contrast, the 2D video learners did not report these problems while using the same head-mounted display. Although the participants might have an adequate cognitive function to overcome the impaired attention for obtaining the H&P competence, the instructors need to design a 360° VR video with less distraction and lower cognitive load. A motion capture unit to synchronize vestibular response and visual information may reduce motion sickness [83], and further studies are warranted to investigate this issue.

Interestingly, the elevated LF/HF ratio at 5 to 10 minutes seemed to be associated with lower hedonic stimulation. Since these cognitive load estimates were not related to Milestone level, they could be used to improve the quality of video learning. For example, the virtual environment sustained the increased LF/HF ratio and acted as a stimulatory driver for cardiac autonomic activity after mental stress [84]. Therefore, limited time to immerse into the virtual environment could improve the learners’ experience.

This study applied the adaptive EMD method and the well-behaved Hilbert transform technique to process the nonstationary electrocardiogram signals [63,64]. Importantly, we found that all RR intervals at different time points significantly and positively correlated with the total cognitive load. Furthermore, the significant association between the RR at 5 to 10 minutes and the Paas-CLS score was independent of the video module. Using similar analytic technology, Ghaderyan and Abbasi also observed that an increased RR interval was associated with higher cognitive load estimations [85]. Moreover, prolonged reaction time at the 5th minute seemed to be related to the RR at 5 to 10 minutes. These observations reflected that novice learners need to improve their cognitive efficiency when learning from dynamically filmed video from a first-person perspective for dealing with elevated cognitive load. These results also indicated that subjective and objective estimates of cognitive load and HRV monitoring have considerable potential to help us better understand a learner’s processing strategy to video learning modules, enhance our comprehension of contextual information, and improve video learning. Future studies are warranted to confirm the benefits of cognitive load and HRV measures in video learning.

### Conclusions

Our preliminary results suggested that 360° VR video learning may result in a better H&P performance despite causing a more prolonged secondary-task reaction time than 2D video learning. Furthermore, the increased LF/HF ratio was associated with lower acceptance of video learning. Qualitative evaluation reflected varying benefits and faults between the 360° VR and 2D videos. Consequently, the students seemed to consume more cognitive resources to fit the immersive 360° VR video than 2D video learning. Without cognitive overload, the undergraduate medical students could adjust their mental efficiency to handle the decreased cognitive reserve when learning through the 360° VR video module. This study indicates that the ubiquitous and diverse roles of multimodal cognitive load estimations and HRV measures in video learning may function as part of an integrated CBME curriculum. Further research is necessary to assess their effects on other performance outcomes.

## Appendix

Multimedia Appendix 1 CONSORT-EHEALTH checklist V1.6.1.

## Abbreviations

CBME: competency-based medical education
EMD: empirical mode decomposition
GEFT: Group Embedded Figures Test
H&P: history taking and physical examination
HF: high frequency
HRV: heart rate variability
LF: low frequency
NASA-TLX: National Aeronautics and Space Administration Task Load Index
ORL-HNS: otorhinolaryngology–head and neck surgery
Paas-CLS: Paas Cognitive Load Scale
RMSSD: root mean square of successive heartbeat interval difference
RR: R wave to R wave
SDB: sleep-disordered breathing
SDNN: SD of normal-to-normal RR intervals
VR: virtual reality

## References

[1] Carraccio C, Wolfsthal SD, Englander R, Ferentz K, Martin C (2002). Shifting paradigms: From Flexner to competencies. Acad Med.

[2] Antonoff MB, Swanson JA, Green CA, Mann BD, Maddaus MA, D'Cunha J (2012). The significant impact of a competency-based preparatory course for senior medical students entering surgical residency. Acad Med.

[3] Abe T, Dar F, Amnattrakul P, Aydin A, Raison N, Shinohara N, Khan MS, Ahmed K, Dasgupta P (2019). The effect of repeated full immersion simulation training in ureterorenoscopy on mental workload of novice operators. BMC Med Educ.

[4] Holmboe ES, Sherbino J, Long DM, Swing SR, Frank JR (2010). The role of assessment in competency-based medical education. Med Teach.

[5] Liaw SY, Wong LF, Lim EYP, Ang SBL, Mujumdar S, Ho JTY, Mordiffi SZ, Ang ENK (2016). Effectiveness of a web-based simulation in improving nurses' workplace practice with deteriorating ward patients: A pre- and postintervention study. J Med Internet Res.

[6] Accreditation Council for Graduate Medical Education (2014). The Otolaryngology Milestone Project. J Grad Med Educ.

[7] Tsue TT (2014). Developing the otolaryngology milestones. J Grad Med Educ.

[8] Boulet JR, McKinley DW, Norcini JJ, Whelan GP (2002). Assessing the comparability of standardized patient and physician evaluations of clinical skills. Adv Health Sci Educ Theory Pract.

[9] Stewart CM, Masood H, Pandian V, Laeeq K, Akst L, Francis HW, Bhatti NI (2010). Development and pilot testing of an objective structured clinical examination (OSCE) on hoarseness. Laryngoscope.

[10] Guze PA (2015). Using technology to meet the challenges of medical education. Trans Am Clin Climatol Assoc.

[11] Kyaw BM, Saxena N, Posadzki P, Vseteckova J, Nikolaou CK, George PP, Divakar U, Masiello I, Kononowicz AA, Zary N, Tudor Car L (2019). Virtual reality for health professions education: Systematic review and meta-analysis by the Digital Health Education Collaboration. J Med Internet Res.

[12] Sukumar S, Zakaria A, Lai CJ, Sakumoto M, Khanna R, Choi N (2021). Designing and implementing a novel virtual rounds curriculum for medical students' internal medicine clerkship during the COVID-19 pandemic. MedEdPORTAL.

[13] Kaufman DM, Bell W (1997). Teaching and assessing clinical skills using virtual reality. Stud Health Technol Inform.

[14] Keller MS, Park HJ, Cunningham ME, Fouladian JE, Chen M, Spiegel BMR (2017). Public perceptions regarding use of virtual reality in health care: A social media content analysis using Facebook. J Med Internet Res.

[15] Samadbeik M, Yaaghobi D, Bastani P, Abhari S, Rezaee R, Garavand A (2018). The applications of virtual reality technology in medical groups teaching. J Adv Med Educ Prof.

[16] Evans CH, Schenarts KD (2016). Evolving educational techniques in surgical training. Surg Clin North Am.

[17] Yoganathan S, Finch DA, Parkin E, Pollard J (2018). 360° virtual reality video for the acquisition of knot tying skills: A randomised controlled trial. Int J Surg.

[18] Hsieh MC, Lin YH (2017). VR and AR applications in medical practice and education [Article in Chinese]. Hu Li Za Zhi.

[19] Hovgaard LH, Andersen SAW, Konge L, Dalsgaard T, Larsen CR (2018). Validity evidence for procedural competency in virtual reality robotic simulation, establishing a credible pass/fail standard for the vaginal cuff closure procedure. Surg Endosc.

[20] Våpenstad C, Hofstad EF, Bø LE, Kuhry E, Johnsen G, Mårvik R, Langø T, Hernes TN (2017). Lack of transfer of skills after virtual reality simulator training with haptic feedback. Minim Invasive Ther Allied Technol.

[21] Andersen SAW, Konge L, Sørensen MS (2018). The effect of distributed virtual reality simulation training on cognitive load during subsequent dissection training. Med Teach.

[22] Mayer RE, Moreno R (2003). Nine ways to reduce cognitive load in multimedia learning. Educ Psychol.

[23] Fraser K, Ma I, Teteris E, Baxter H, Wright B, McLaughlin K (2012). Emotion, cognitive load and learning outcomes during simulation training. Med Educ.

[24] Frederiksen JG, Sørensen SMD, Konge L, Svendsen MBS, Nobel-Jørgensen M, Bjerrum F, Andersen SAW (2020). Cognitive load and performance in immersive virtual reality versus conventional virtual reality simulation training of laparoscopic surgery: A randomized trial. Surg Endosc.

[25] Sankaranarayanan G, Odlozil CA, Wells KO, Leeds SG, Chauhan S, Fleshman JW, Jones DB, De S (2020). Training with cognitive load improves performance under similar conditions in a real surgical task. Am J Surg.

[26] van Merriënboer JJG, Sweller J (2010). Cognitive load theory in health professional education: Design principles and strategies. Med Educ.

[27] Young JQ, Sewell JL (2015). Applying cognitive load theory to medical education: Construct and measurement challenges. Perspect Med Educ.

[28] Paas FG (1992). Training strategies for attaining transfer of problem-solving skill in statistics: A cognitive-load approach. J Educ Psychol.

[29] Leppink J, Paas F, van Gog T, van der Vleuten CP, van Merriënboer JJG (2014). Effects of pairs of problems and examples on task performance and different types of cognitive load. Learn Instr.

[30] Naismith LM, Cheung JJH, Ringsted C, Cavalcanti RB (2015). Limitations of subjective cognitive load measures in simulation-based procedural training. Med Educ.

[31] Paas F, Renkl A, Sweller J (2003). Cognitive load theory and instructional design: Recent developments. Educ Psychol.

[32] Ginns P (2006). Integrating information: A meta-analysis of the spatial contiguity and temporal contiguity effects. Learn Instr.

[33] Georgsson M (2019). NASA RTLX as a novel assessment for determining cognitive load and user acceptance of expert and user-based evaluation methods exemplified through a mHealth diabetes self-management application evaluation. Stud Health Technol Inform.

[34] Rölfing JD, Nørskov JK, Paltved C, Konge L, Andersen SAW (2019). Failure affects subjective estimates of cognitive load through a negative carry-over effect in virtual reality simulation of hip fracture surgery. Adv Simul (Lond).

[35] Haji FA, Khan R, Regehr G, Drake J, de Ribaupierre S, Dubrowski A (2015). Measuring cognitive load during simulation-based psychomotor skills training: Sensitivity of secondary-task performance and subjective ratings. Adv Health Sci Educ Theory Pract.

[36] Mandrick K, Peysakhovich V, Rémy F, Lepron E, Causse M (2016). Neural and psychophysiological correlates of human performance under stress and high mental workload. Biol Psychol.

[37] Künstler ECS, Penning MD, Napiórkowski N, Klingner CM, Witte OW, Müller HJ, Bublak P, Finke K (2018). Dual task effects on visual attention capacity in normal aging. Front Psychol.

[38] Fadeev KA, Smirnov AS, Zhigalova OP, Bazhina PS, Tumialis AV, Golokhvast KS (2020). Too real to be virtual: Autonomic and EEG responses to extreme stress scenarios in virtual reality. Behav Neurol.

[39] Task Force of the European Society of Cardiology and the North American Society of Pacing and Electrophysiology (1996). Heart rate variability: Standards of measurement, physiological interpretation and clinical use. Circulation.

[40] Kim HG, Cheon EJ, Bai DS, Lee YH, Koo BH (2018). Stress and heart rate variability: A meta-analysis and review of the literature. Psychiatry Investig.

[41] Ham J, Cho D, Oh J, Lee B (2017). Discrimination of multiple stress levels in virtual reality environments using heart rate variability. Proceedings of the 39th Annual International Conference of the IEEE Engineering in Medicine and Biology Society.

[42] McDuff D, Gontarek S, Picard R (2014). Remote measurement of cognitive stress via heart rate variability. Proceedings of the 36th Annual International Conference of the IEEE Engineering in Medicine and Biology Society.

[43] Fuentes-García JP, Villafaina S, Collado-Mateo D, de la Vega R, Olivares PR, Clemente-Suárez VJ (2019). Differences between high vs low performance chess players in heart rate variability during chess problems. Front Psychol.

[44] Bergström I, Kilteni K, Slater M (2016). First-person perspective virtual body posture influences stress: A virtual reality body ownership study. PLoS One.

[45] Ohyama S, Nishiike S, Watanabe H, Matsuoka K, Akizuki H, Takeda N, Harada T (2007). Autonomic responses during motion sickness induced by virtual reality. Auris Nasus Larynx.

[46] Nasca TJ, Philibert I, Brigham T, Flynn TC (2012). The next GME accreditation system--Rationale and benefits. N Engl J Med.

[47] Hassenzahl M, Burmester M, Koller F, Ziegler J, Szwillus G (2003). AttrakDiffin fragebogen zur messung wahrgenommener hedonischer und pragmatischer qualität. Mensch & Computer.

[48] Lee LA, Wang SL, Chao YP, Tsai MS, Hsin LJ, Kang CJ, Fu CH, Chao WC, Huang CG, Li HY, Chuang CK (2018). Mobile technology in e-learning for undergraduate medical education on emergent otorhinolaryngology-head and neck surgery disorders: Pilot randomized controlled trial. JMIR Med Educ.

[49] Noll C, von Jan U, Raap U, Albrecht UV (2017). Mobile augmented reality as a feature for self-oriented, blended learning in medicine: Randomized controlled trial. JMIR Mhealth Uhealth.

[50] Eysenbach G, CONSORT-EHEALTH Group (2011). CONSORT-EHEALTH: Improving and standardizing evaluation reports of web-based and mobile health interventions. J Med Internet Res.

[51] Morrison GR, Ross SM, Kalman HK, Kemp JE (2013). Designing Effective Instruction. 7th edition.

[52] Renkl A, Stark R, Gruber H, Mandl H (1998). Learning from worked-out examples: The effects of example variability and elicited self-explanations. Contemp Educ Psychol.

[53] Chi MTH, Bassok M, Lewis MW, Reimann P, Glaser R (1989). Self-explanations: How students study and use examples in learning to solve problems. Cogn Sci.

[54] Scott N, Smith DU, Rosenberg IK (1981). Cognitive style and instructional materials for medical students. J Med Educ.

[55] Chapman DM, Calhoun JG (2006). Validation of learning style measures: Implications for medical education practice. Med Educ.

[56] Lee LA, Chao YP, Huang CG, Fang JT, Wang SL, Chuang CK, Kang CJ, Hsin LJ, Lin WN, Fang TJ, Li HY (2018). Cognitive style and mobile e-learning in emergent otorhinolaryngology-head and neck surgery disorders for millennial undergraduate medical students: Randomized controlled trial. J Med Internet Res.

[57] Witkin HA, Oltman PK, Raskin E, Karp SA (1971). A Manual for the Embedded Figures Tests.

[58] Paas FG, Van Merriënboer JJ, Adam JJ (1994). Measurement of cognitive load in instructional research. Percept Mot Skills.

[59] Xiao YM, Wang ZM, Wang MZ, Lan YJ (2005). The appraisal of reliability and validity of subjective workload assessment technique and NASA-Task Load Index [Article in Chinese]. Zhonghua Lao Dong Wei Sheng Zhi Ye Bing Za Zhi.

[60] Carswell CM, Clarke D, Seales WB (2005). Assessing mental workload during laparoscopic surgery. Surg Innov.

[61] Guo J, Dai Y, Wang C, Wu H, Xu T, Lin K (2019). A physiological data‐driven model for learners' cognitive load detection using HRV‐PRV feature fusion and optimized XGBoost classification. J Softw.

[62] Fuentes-García JP, Pereira T, Castro MA, Carvalho Santos A, Villafaina S (2019). Psychophysiological stress response of adolescent chess players during problem-solving tasks. Physiol Behav.

[63] Huang NE, Shen Z, Long SR, Wu MC, Shih HH, Zheng Q, Yen N, Tung CC, Liu HH (1998). The empirical mode decomposition and the Hilbert spectrum for nonlinear and non-stationary time series analysis. Proc R Soc Lond A Math Phys Sci.

[64] Souza Neto EP, Custaud MA, Cejka JC, Abry P, Frutoso J, Gharib C, Flandrin P (2004). Assessment of cardiovascular autonomic control by the empirical mode decomposition. Methods Inf Med.

[65] Task Force of the European Society of Cardiology and the North American Society of Pacing and Electrophysiology (1996). Heart rate variability. Standards of measurement, physiological interpretation, and clinical use. Eur Heart J.

[66] Shaffer F, Ginsberg JP (2017). An overview of heart rate variability metrics and norms. Front Public Health.

[67] Solhjoo S, Haigney MC, McBee E, van Merrienboer JJG, Schuwirth L, Artino AR, Battista A, Ratcliffe TA, Lee HD, Durning SJ (2019). Heart rate and heart rate variability correlate with clinical reasoning performance and self-reported measures of cognitive load. Sci Rep.

[68] Suriya-Prakash M, John-Preetham G, Sharma R (2017). Does heart rate variability predict human cognitive performance at higher memory loads?. Indian J Physiol Pharmacol.

[69] Hansen AL, Johnsen BH, Sollers JJ 3rd, Stenvik K, Thayer JF (2004). Heart rate variability and its relation to prefrontal cognitive function: The effects of training and detraining. Eur J Appl Physiol.

[70] Durantin G, Gagnon JF, Tremblay S, Dehais F (2014). Using near infrared spectroscopy and heart rate variability to detect mental overload. Behav Brain Res.

[71] Ingadottir B, Blondal K, Thue D, Zoega S, Thylen I, Jaarsma T (2017). Development, usability, and efficacy of a serious game to help patients learn about pain management after surgery: An evaluation study. JMIR Serious Games.

[72] Armstrong RA (2014). When to use the Bonferroni correction. Ophthalmic Physiol Opt.

[73] Perneger TV (1998). What's wrong with Bonferroni adjustments. BMJ.

[74] Hansen M, Oosthuizen G, Windsor J, Doherty I, Greig S, McHardy K, McCann L (2011). Enhancement of medical interns' levels of clinical skills competence and self-confidence levels via video iPods: Pilot randomized controlled trial. J Med Internet Res.

[75] Bochenska K, Milad MP, DeLancey JO, Lewicky-Gaupp C (2018). Instructional video and medical student surgical knot-tying proficiency: Randomized controlled trial. JMIR Med Educ.

[76] Walthouwer MJ, Oenema A, Lechner L, de Vries H (2015). Comparing a video and text version of a web-based computer-tailored intervention for obesity prevention: A randomized controlled trial. J Med Internet Res.

[77] Azer SA, Algrain HA, AlKhelaif RA, AlEshaiwi SM (2013). Evaluation of the educational value of YouTube videos about physical examination of the cardiovascular and respiratory systems. J Med Internet Res.

[78] Lambooij M, IJsselsteijn W, Fortuin M, Heynderickx I (2009). Visual discomfort and visual fatigue of stereoscopic displays: A review. J Imaging Sci Technol.

[79] Moss JD, Muth ER (2011). Characteristics of head-mounted displays and their effects on simulator sickness. Hum Factors.

[80] Xu W, Liang HN, Zhang Z, Baghaei N (2020). Studying the effect of display type and viewing perspective on user experience in virtual reality exergames. Games Health J.

[81] Park S, Won MJ, Mun S, Lee EC, Whang M (2014). Does visual fatigue from 3D displays affect autonomic regulation and heart rhythm?. Int J Psychophysiol.

[82] Carnegie K, Rhee T (2015). Reducing visual discomfort with HMDs using dynamic depth of field. IEEE Comput Graph Appl.

[83] Byagowi A, Mohaddes D, Moussavi Z (2014). Design and application of a novel virtual reality navigational technology (VRNChair). J Exp Neurosci.

[84] Kazzi C, Blackmore C, Shirbani F, Tan I, Butlin M, Avolio AP, Barin E (2018). Effects of instructed meditation augmented by computer-rendered artificial virtual environment on heart rate variability. Proceedings of the 40th Annual International Conference of the IEEE Engineering in Medicine and Biology Society.

[85] Ghaderyan P, Abbasi A (2017). Dynamic Hilbert warping, a new measure of RR-interval signals evaluated in the cognitive load estimation. Med Eng Phys.

