# Supplementary material for: Using a 360° Virtual Reality or 2D Video to Learn History Taking and Physical Examination Skills for Undergraduate Medical Students: Pilot Randomized Controlled Trial
Source: JMIR Serious Games. 2021 Nov 22;9(4):e13124. doi: 10.2196/13124 (PMC8663656; doi:10.2196/13124)
Supplement: Multimedia Appendix 1 [file games_v9i4e13124_app1.pdf]

CONSORT-EHEALTH (V 1.6.1) - Submission/Publication Form

The CONSORT-EHEALTH checklist is intended for authors of randomized trials evaluating web-based and Internet-based applications/interventions, including mobile interventions, electronic games (incl multiplayer games), social media, certain telehealth applications, and other interactive and/or networked electronic applications. Some of the items (e.g. all subitems under item 5 - description of the intervention) may also be applicable for other study designs.

The goal of the CONSORT EHEALTH checklist and guideline is to be
a) a guide for reporting for authors of RCTs,
b) to form a basis for appraisal of an ehealth trial (in terms of validity)

CONSORT-EHEALTH items/subitems are MANDATORY reporting items for studies published in the Journal of Medical Internet Research and other journals / scientific societies endorsing the checklist.

Items numbered 1., 2., 3., 4a., 4b etc are original CONSORT or CONSORT-NPT (non-pharmacologic treatment) items.
Items with Roman numerals (i., ii, iii, iv etc.) are CONSORT-EHEALTH extensions/clarifications.

As the CONSORT-EHEALTH checklist is still considered in a formative stage, we would ask that you also RATE ON A SCALE OF 1-5 how important/useful you feel each item is FOR THE PURPOSE OF THE CHECKLIST and reporting guideline (optional).

Mandatory reporting items are marked with a red *.
In the textboxes, either copy & paste the relevant sections from your manuscript into this form - please include any quotes from your manuscript in QUOTATION MARKS,
or answer directly by providing additional information not in the manuscript, or elaborating on why the item was not relevant for this study.

YOUR ANSWERS WILL BE PUBLISHED AS A SUPPLEMENTARY FILE TO YOUR PUBLICATION IN JMIR AND ARE CONSIDERED PART OF YOUR PUBLICATION (IF ACCEPTED).
Please fill in these questions diligently. Information will not be copyedited, so please use proper spelling and grammar, use correct capitalization, and avoid abbreviations.

DO NOT FORGET TO SAVE AS PDF _AND_ CLICK THE SUBMIT BUTTON SO YOUR ANSWERS ARE IN OUR DATABASE !!!

Citation Suggestion (if you append the pdf as Appendix we suggest to cite this paper in the caption):
Eysenbach G, CONSORT-EHEALTH Group
[illegible]

你正在編輯自己的回覆內容。只要與他人共用這個網址，就能允許其他使用者編輯你的回覆內容。 填寫新的回覆

URL: [illegible]

doi: 10.2196/jmir.1923
PMID: 22209829

leeleon3855@gmail.com (未分享) 切換帳戶
必須重新提交才能儲存

*必填

Your name *
First Last

Li-Ang Lee

Primary Affiliation (short), City, Country *
University of Toronto, Toronto, Canada

Chang Gung Memorial Hospital, Linkou Main B

Your e-mail address *
abc@gmail.com

5738@cgmh.org.tw

Title of your manuscript *
Provide the (draft) title of your manuscript.

Using A 360° Virtual Reality or Two-Dimensional Video to Learn History Taking and Physical Examination Skills: Pilot Randomized Controlled Trial

Name of your App/Software/Intervention *
If there is a short and a long/alternate name, write the short name first and add the long name in brackets.

你正在編輯自己的回覆內容。只要與他人共用這個網址，就能允許其他使用者編輯你的回覆內容。 填寫新的回覆

Evaluated Version (if any)

e.g. "V1", "Release 2017-03-01", "Version 2.0.27913"

您的回答

Language(s) *

What language is the intervention/app in? If multiple languages are available, separate by comma (e.g. "English, French")

Chinese

URL of your Intervention Website or App

e.g. a direct link to the mobile app on app in appstore (itunes, Google Play), or URL of the website. If the intervention is a DVD or hardware, you can also link to an Amazon page.

https://www.amazon.com/HTC-VIVE-Virtual-Reality-System-pc/dp/B00VF5NT4I

URL of an image/screenshot (optional)

您的回答

Accessibility *

Can an enduser access the intervention presently?

- ○ access is free and open
- ◉ access only for special usergroups, not open
- ○ access is open to everyone, but requires payment/subscription/in-app purchases
- ○ app/intervention no longer accessible
- ○ 其他：

你正在編輯自己的回覆內容。只要與他人共用這個網址，就能允許其他使用者編輯你的回覆內容。 填寫新的回覆

Primary Medical Indication/Disease/Condition *

e.g. "Stress", "Diabetes", or define the target group in brackets after the condition, e.g. "Autism (Parents of children with)", "Alzheimers (Informal Caregivers of)"

Medical Education

Primary Outcomes measured in trial *

comma-separated list of primary outcomes reported in the trial

Milestone level

Secondary/other outcomes

Are there any other outcomes the intervention is expected to affect?

AttrakDiff2 questionnaire scales

Recommended "Dose" *

What do the instructions for users say on how often the app should be used?

- ○ Approximately Daily
- ○ Approximately Weekly
- ○ Approximately Monthly
- ○ Approximately Yearly
- ◉ "as needed"
- ○ 其他:

你正在編輯自己的回覆內容。只要與他人共用這個網址，就能允許其他使用者編輯你的回覆內容。 填寫新的回覆

Approx. Percentage of Users (starters) still using the app as recommended after 3 months *

- (selected) unknown / not evaluated
- 0-10%
- 11-20%
- 21-30%
- 31-40%
- 41-50%
- 51-60%
- 61-70%
- 71%-80%
- 81-90%
- 91-100%
- 其他:

Overall, was the app/intervention effective? *

- yes: all primary outcomes were significantly better in intervention group vs control
- (selected) partly: SOME primary outcomes were significantly better in intervention group vs control
- no statistically significant difference between control and intervention
- potentially harmful: control was significantly better than intervention in one or more outcomes
- inconclusive: more research is needed
- 其他:

你正在編輯自己的回覆內容。只要與他人共用這個網址，就能允許其他使用者編輯你的回覆內容。 填寫新的回覆

Article Preparation Status/Stage *

At which stage in your article preparation are you currently (at the time you fill in this form)

- ○ not submitted yet - in early draft status
- ○ not submitted yet - in late draft status, just before submission
- ○ submitted to a journal but not reviewed yet
- ○ submitted to a journal and after receiving initial reviewer comments
- ◉ submitted to a journal and accepted, but not published yet
- ○ published
- ○ 其他:

Journal *

If you already know where you will submit this paper (or if it is already submitted), please provide the journal name (if it is not JMIR, provide the journal name under "other")

- ○ not submitted yet / unclear where I will submit this
- ○ Journal of Medical Internet Research (JMIR)
- ○ JMIR mHealth and UHealth
- ◉ JMIR Serious Games
- ○ JMIR Mental Health
- ○ JMIR Public Health
- ○ JMIR Formative Research
- ○ Other JMIR sister journal
- ○ 其他:

你正在編輯自己的回覆內容。只要與他人共用這個網址，就能允許其他使用者編輯你的回覆內容。 填寫新的回覆

Is this a full powered effectiveness trial or a pilot/feasibility trial? *

- (x) Pilot/feasibility
- ( ) Fully powered

Manuscript tracking number *

If this is a JMIR submission, please provide the manuscript tracking number under "other" (The ms tracking number can be found in the submission acknowledgement email, or when you login as author in JMIR. If the paper is already published in JMIR, then the ms tracking number is the four-digit number at the end of the DOI, to be found at the bottom of each published article in JMIR)

- ( ) no ms number (yet) / not (yet) submitted to / published in JMIR
- (x) 其他: JSG Manuscript #13124

TITLE AND ABSTRACT

1a) TITLE: Identification as a randomized trial in the title

1a) Does your paper address CONSORT item 1a? *

I.e does the title contain the phrase "Randomized Controlled Trial"? (if not, explain the reason under "other")

- (x) yes
- ( ) 其他:

你正在編輯自己的回覆內容。只要與他人共用這個網址，就能允許其他使用者編輯你的回覆內容。 填寫新的回覆

1a-i) Identify the mode of delivery in the title

Identify the mode of delivery. Preferably use “web-based” and/or “mobile” and/or “electronic game” in the title. Avoid ambiguous terms like “online”, “virtual”, “interactive”. Use “Internet-based” only if Intervention includes non-web-based Internet components (e.g. email), use “computer-based” or “electronic” only if offline products are used. Use “virtual” only in the context of “virtual reality” (3-D worlds). Use “online” only in the context of “online support groups”. Complement or substitute product names with broader terms for the class of products (such as “mobile” or “smart phone” instead of “iphone”), especially if the application runs on different platforms.

	1	2	3	4	5	
subitem not at all important	○	○	○	◉	○	essential

清除選取的項目

Does your paper address subitem 1a-i? *

Copy and paste relevant sections from manuscript title (include quotes in quotation marks "like this" to indicate direct quotes from your manuscript), or elaborate on this item by providing additional information not in the ms, or briefly explain why the item is not applicable/relevant for your study

Using A "360° Virtual Reality" or Two-Dimensional Video to Learn History Taking and Physical Examination Skills for Undergraduate Medical Students: Pilot Randomized Controlled Trial

1a-ii) Non-web-based components or important co-interventions in title

Mention non-web-based components or important co-interventions in title, if any (e.g., “with telephone support”).

	1	2	3	4	5	
subitem not at all important	○	○	○	◉	○	essential

清除選取的項目

你正在編輯自己的回覆內容。只要與他人共用這個網址，就能允許其他使用者編輯你的回覆內容。 填寫新的回覆

Does your paper address subitem 1a-ii?

Copy and paste relevant sections from manuscript title (include quotes in quotation marks "like this" to indicate direct quotes from your manuscript), or elaborate on this item by providing additional information not in the ms, or briefly explain why the item is not applicable/relevant for your study

Using A 360° Virtual Reality or "Two-Dimensional Video" to Learn History Taking and Physical Examination Skills for Undergraduate Medical Students: Pilot Randomized Controlled Trial

1a-iii) Primary condition or target group in the title

Mention primary condition or target group in the title, if any (e.g., "for children with Type I Diabetes") Example: A Web-based and Mobile Intervention with Telephone Support for Children with Type I Diabetes: Randomized Controlled Trial

	1	2	3	4	5	
subitem not at all important	○	○	○	○	◉	essential

清除選取的項目

Does your paper address subitem 1a-iii? *

Copy and paste relevant sections from manuscript title (include quotes in quotation marks "like this" to indicate direct quotes from your manuscript), or elaborate on this item by providing additional information not in the ms, or briefly explain why the item is not applicable/relevant for your study

Using A 360° Virtual Reality or Two-Dimensional Video to Learn History Taking and Physical Examination Skills "for Undergraduate Medical Students": Pilot Randomized Controlled Trial

1b) ABSTRACT: Structured summary of trial design, methods, results, and conclusions

NPT extension: Description of experimental treatment, comparator, care providers, centers, and blinding status.

你正在編輯自己的回覆內容。只要與他人共用這個網址，就能允許其他使用者編輯你的回覆內容。

填寫新的回覆

1b-i) Key features/functionalities/components of the intervention and comparator in the METHODS section of the ABSTRACT

Mention key features/functionalities/components of the intervention and comparator in the abstract. If possible, also mention theories and principles used for designing the site. Keep in mind the needs of systematic reviewers and indexers by including important synonyms. (Note: Only report in the abstract what the main paper is reporting. If this information is missing from the main body of text, consider adding it)

	1	2	3	4	5	
subitem not at all important	○	○	○	○	◉	essential

清除選取的項目

Does your paper address subitem 1b-i? *

Copy and paste relevant sections from the manuscript abstract (include quotes in quotation marks "like this" to indicate direct quotes from your manuscript), or elaborate on this item by providing additional information not in the ms, or briefly explain why the item is not applicable/relevant for your study

The students were randomly assigned (1:1) to a 360° VR video group and a 2-D video group matched by age, sex, and cognitive style. The contents of both videos were different with regards to visual angle and self-determination.

1b-ii) Level of human involvement in the METHODS section of the ABSTRACT

Clarify the level of human involvement in the abstract, e.g., use phrases like “fully automated” vs. “therapist/nurse/care provider/physician-assisted” (mention number and expertise of providers involved, if any). (Note: Only report in the abstract what the main paper is reporting. If this information is missing from the main body of text, consider adding it)

	1	2	3	4	5	
subitem not at all important	○	○	◉	○	○	essential

清除選取的項目

你正在編輯自己的回覆內容。只要與他人共用這個網址，就能允許其他使用者編輯你的回覆內容。 填寫新的回覆

Does your paper address subitem 1b-ii?

Copy and paste relevant sections from the manuscript abstract (include quotes in quotation marks "like this" to indicate direct quotes from your manuscript), or elaborate on this item by providing additional information not in the ms, or briefly explain why the item is not applicable/relevant for your study

This pilot system-design study included 32 undergraduate medical students at an academic teaching hospital.

1b-iii) Open vs. closed, web-based (self-assessment) vs. face-to-face assessments in the METHODS section of the ABSTRACT

Mention how participants were recruited (online vs. offline), e.g., from an open access website or from a clinic or a closed online user group (closed usergroup trial), and clarify if this was a purely web-based trial, or there were face-to-face components (as part of the intervention or for assessment). Clearly say if outcomes were self-assessed through questionnaires (as common in web-based trials). Note: In traditional offline trials, an open trial (open-label trial) is a type of clinical trial in which both the researchers and participants know which treatment is being administered. To avoid confusion, use “blinded” or “unblinded” to indicated the level of blinding instead of “open”, as “open” in web-based trials usually refers to “open access” (i.e. participants can self-enrol). (Note: Only report in the abstract what the main paper is reporting. If this information is missing from the main body of text, consider adding it)

	1	2	3	4	5	
subitem not at all important	○	○	○	●	○	essential

清除選取的項目

Does your paper address subitem 1b-iii?

Copy and paste relevant sections from the manuscript abstract (include quotes in quotation marks "like this" to indicate direct quotes from your manuscript), or elaborate on this item by providing additional information not in the ms, or briefly explain why the item is not applicable/relevant for your study

Subjective and objective cognitive loads were estimated using the Paas Cognitive Load Scale, NASA Task Load Index, and secondary-task reaction time, respectively. Cardiac autonomic function was assessed using HRV measurements. Learning experience was assessed using the AttrakDiff2 questionnaire and qualitative feedback.

你正在編輯自己的回覆內容。只要與他人共用這個網址，就能允許其他使用者編輯你的回覆內容。 填寫新的回覆

1b-iv) RESULTS section in abstract must contain use data

Report number of participants enrolled/assessed in each group, the use/uptake of the intervention (e.g., attrition/adherence metrics, use over time, number of logins etc.), in addition to primary/secondary outcomes. (Note: Only report in the abstract what the main paper is reporting. If this information is missing from the main body of text, consider adding it)

	1	2	3	4	5	
subitem not at all important	○	○	○	○	◉	essential

清除選取的項目

Does your paper address subitem 1b-iv?

Copy and paste relevant sections from the manuscript abstract (include quotes in quotation marks "like this" to indicate direct quotes from your manuscript), or elaborate on this item by providing additional information not in the ms, or briefly explain why the item is not applicable/relevant for your study

All of the participants (20 [62%] males and 12 [38%] females; median age 24 years, interquartile range 23–25 years) received the intended intervention. The 360° VR video group seemed to have a higher Milestone level than the 2-D video group (P=.04). The reaction time at the 10th min in the 360° VR video group was significantly higher than that in the 2D video group (P<.001). Multiple logistic regression models of the overall cohort showed that the 360° VR video module was independently and positively associated with a reaction time at the 10th min ≥3.6 sec (exp B=18.8, 95% CI 3.2–110.8, P=.001) and Milestone level ≥3 (exp B=15.0, 95% confidence interval [CI] 2.3–99.6, P=.005). However, a reaction time at the 10th min ≥3.6 sec was not related to Milestone level ≥3. A low frequency/high frequency ratio between the 5th and 10th min ≥1.43 seemed to be inversely associated with a hedonic stimulation score ≥2.0 (exp B=0.14, 95% CI 0.03–0.68, P=.015) after adjusting for the video module.

1b-v) CONCLUSIONS/DISCUSSION in abstract for negative trials

Conclusions/Discussions in abstract for negative trials: Discuss the primary outcome - if the trial is negative (primary outcome not changed), and the intervention was not used, discuss whether negative results are attributable to lack of uptake and discuss reasons. (Note: Only report in the abstract what the main paper is reporting. If this information is missing from the main body of text, consider adding it)

	1	2	3	4	5	
subitem not at all important	○	○	○	◉	○	essential

你正在編輯自己的回覆內容。只要與他人共用這個網址，就能允許其他使用者編輯你的回覆內容。

填寫新的回覆

Does your paper address subitem 1b-v?

Copy and paste relevant sections from the manuscript abstract (include quotes in quotation marks "like this" to indicate direct quotes from your manuscript), or elaborate on this item by providing additional information not in the ms, or briefly explain why the item is not applicable/relevant for your study

Our preliminary results showed that 360° VR video learning may be associated with a better Milestone level than 2-D video learning, and that this did not seem to be related to cognitive load estimates or HRV indexes in the novice learners. Of note, an increase in sympathovagal balance may have been associated with a lower hedonic stimulation score, which may have met the learners' needs and prompted learning through the different video modules.

INTRODUCTION

2a) In INTRODUCTION: Scientific background and explanation of rationale

2a-i) Problem and the type of system/solution

Describe the problem and the type of system/solution that is object of the study: intended as stand-alone intervention vs. incorporated in broader health care program? Intended for a particular patient population? Goals of the intervention, e.g., being more cost-effective to other interventions, replace or complement other solutions? (Note: Details about the intervention are provided in "Methods" under 5)

	1	2	3	4	5	
subitem not at all important	○	○	○	◉	○	essential

清除選取的項目

你正在編輯自己的回覆內容。只要與他人共用這個網址，就能允許其他使用者編輯你的回覆內容。 填寫新的回覆

Does your paper address subitem 2a-i? *

Copy and paste relevant sections from the manuscript (include quotes in quotation marks "like this" to indicate direct quotes from your manuscript), or elaborate on this item by providing additional information not in the ms, or briefly explain why the item is not applicable/relevant for your study

CBME is used to improve graduates' competency levels, ensuring they are skillful and qualified in all critical areas of their occupation. To this end, CBME emphasizes the use and importance of simulation-based training, which considers patient safety and for which real-life opportunities are limited [5]. This consideration is crucial for medical learners and teachers during the coronavirus disease-2019 (COVID-19) pandemic. History taking and physical examination (H&P) is a principal competency, incorporating knowledge, skills, and behavior to initially approach a patient. Therefore, H&P is a key performance level of otorhinolaryngology-head and neck surgery (ORL-HNS) [6]. To enhance the development of H&P skills, commonly used methods to assess this competency include in-training examinations such as the Milestone [7], Mini-Clinical Evaluation Exercise [8], and oral examinations of clinical practice [9]. Recently, simulations such as part-time trainers, integrated simulators, virtual reality (VR), and wearable devices have become increasingly popular for CBME [10]. Simulation-based training, such as VR, has been shown to improve health professionals' knowledge and skills outcomes and be an integrative step towards supervised clinical practice [11]. Especially, VR allowed students to practice rounding skills, facilitate education during the COVID-19 pandemic, and supplement in-person clerkship education [12].

2a-ii) Scientific background, rationale: What is known about the (type of) system

Scientific background, rationale: What is known about the (type of) system that is the object of the study (be sure to discuss the use of similar systems for other conditions/diagnoses, if appropiate), motivation for the study, i.e. what are the reasons for and what is the context for this specific study, from which stakeholder viewpoint is the study performed, potential impact of findings [2]. Briefly justify the choice of the comparator.

	1	2	3	4	5	
subitem not at all important	○	○	○	◉	○	essential

清除選取的項目

你正在編輯自己的回覆內容。只要與他人共用這個網址，就能允許其他使用者編輯你的回覆內容。

填寫新的回覆

Does your paper address subitem 2a-ii? *

Copy and paste relevant sections from the manuscript (include quotes in quotation marks "like this" to indicate direct quotes from your manuscript), or elaborate on this item by providing additional information not in the ms, or briefly explain why the item is not applicable/relevant for your study

VR consists of a computer-generated three-dimensional (3-D) simulation in which the user both explores and manipulates the contents of the environment to learn and assess clinical knowledge and skills [13]. VR provides experiential learning and provides standardized, controlled exposure to situated events, patient-caregiver communication, and teamwork. The use of VR has been shown to be highly acceptable by learners in a wide range of health care settings [14] and to play an essential role in improving performance [15]. 360° VR video is a subtype of image-based VR and represents an immersive, 3-D medium featuring authenticity and fidelity using a VR head-mounted display. 360° VR video opens up many possibilities in many domains of medical education [16], such as an independent teaching aid or an adjunct to traditional face-to-face teaching [17]. The application of 360° VR video has enhanced the effectiveness of medical education and training, raised the level of diagnosis and treatment, improved the doctor-patient relationship, and boosted medical execution efficiency [18]. Using 360° VR videos to facilitate the acquisition of new clinical skills has been suggested to be a valuable step in developing a clinical teaching curriculum [19].

2b) In INTRODUCTION: Specific objectives or hypotheses

Does your paper address CONSORT subitem 2b? *

Copy and paste relevant sections from the manuscript (include quotes in quotation marks "like this" to indicate direct quotes from your manuscript), or elaborate on this item by providing additional information not in the ms, or briefly explain why the item is not applicable/relevant for your study

The present study aimed (1) to evaluate differences between two learning materials (360° VR video and 2-D video) in the subjective and objective cognitive loads, autonomic function, outcome, and learning experience while they were learning H&P skills and (2) to study how learning materials (360° VR or 2-D video) impact learning outcomes through the changes in cognitive load and HRV. The initial hypotheses were that (1) subjective and objective cognitive loads and autonomic function would be changed while using different learning materials, and (2) learners with higher learning outcome and experience would have different cognitive load and autonomic function during the learning tasks than the low learning outcome and experience.

METHODS

你正在編輯自己的回覆內容。只要與他人共用這個網址，就能允許其他使用者編輯你的回覆內容。 填寫新的回覆

3a) Description of trial design (such as parallel, factorial) including allocation ratio

Does your paper address CONSORT subitem 3a? *

Copy and paste relevant sections from the manuscript (include quotes in quotation marks "like this" to indicate direct quotes from your manuscript), or elaborate on this item by providing additional information not in the ms, or briefly explain why the item is not applicable/relevant for your study

We conducted a prospective, randomized controlled, pilot system-design study from 1 June 2018 to 30 October 2018 at an academic teaching hospital (Department of ORL-HNS, Linkou Chang Gung Memorial Hospital, Taoyuan, Taiwan). The participants were blinded to the purpose of the study during recruitment to minimize preparation bias. After the participants had provided consent and completed the GEFT, we randomly assigned them (1:1) to the 360° VR video group and 2-D video group matched by age, sex, and cognitive style (Figure 1).

3b) Important changes to methods after trial commencement (such as eligibility criteria), with reasons

Does your paper address CONSORT subitem 3b? *

Copy and paste relevant sections from the manuscript (include quotes in quotation marks "like this" to indicate direct quotes from your manuscript), or elaborate on this item by providing additional information not in the ms, or briefly explain why the item is not applicable/relevant for your study

No, we did not change to methods.

3b-i) Bug fixes, Downtimes, Content Changes

Bug fixes, Downtimes, Content Changes: ehealth systems are often dynamic systems. A description of changes to methods therefore also includes important changes made on the intervention or comparator during the trial (e.g., major bug fixes or changes in the functionality or content) (5-iii) and other “unexpected events” that may have influenced study design such as staff changes, system failures/downtimes, etc. [2].

	1	2	3	4	5	
subitem not at all important	○	○	○	◉	○	essential

你正在編輯自己的回覆內容。只要與他人共用這個網址，就能允許其他使用者編輯你的回覆內容。 填寫新的回覆

Does your paper address subitem 3b-i?

Copy and paste relevant sections from the manuscript (include quotes in quotation marks "like this" to indicate direct quotes from your manuscript), or elaborate on this item by providing additional information not in the ms, or briefly explain why the item is not applicable/relevant for your study

We then developed courseware with the same user interface for the 360° VR and 2-D videos using Unity 2017.3.1 Editor (Unity Technologies, San Francisco, CA, United States).

4a) Eligibility criteria for participants

Does your paper address CONSORT subitem 4a? *

Copy and paste relevant sections from the manuscript (include quotes in quotation marks "like this" to indicate direct quotes from your manuscript), or elaborate on this item by providing additional information not in the ms, or briefly explain why the item is not applicable/relevant for your study

A convenience sampling approach was used, and 32 consecutive volunteers were recruited during the study period. The inclusion criteria were as follows: (1) age >20 years, and (2) novices in ORL-HNS (undergraduate medical students). The exclusion criteria were: 1) contraindications for using VR (such as pregnancy, hypertension, motion sickness, inner ear infections or claustrophobia, recent surgery, pre-existing binocular vision abnormalities, heart disorders, or epilepsy), and (2) declining to participate. All of the volunteers had at least a basic level of computer literacy and could use VR headsets and controllers after instruction. We used the 25-item Group Embedded Figures Test (GEFT) (score range, 0–18) to assess the participants' cognitive style [54]. The GEFT has been shown to have high reliability in medical education [55], and we have previously validated its effectiveness of classifying learning preference in millennial undergraduate medical students [56]. Field-independent learners prefer and have better performance in computer-assisted learning. We stratified the students into two subgroups: "field-dependence" (GEFT score ≤12) and "field-independence" (GEFT score >12) [57].

4a-i) Computer / Internet literacy

Computer / Internet literacy is often an implicit "de facto" eligibility criterion - this should be explicitly clarified.

	1	2	3	4	5	
subitem not at all important	○	○	○	◉	○	essential

你正在編輯自己的回覆內容。只要與他人共用這個網址，就能允許其他使用者編輯你的回覆內容。 填寫新的回覆

Does your paper address subitem 4a-i?

Copy and paste relevant sections from the manuscript (include quotes in quotation marks "like this" to indicate direct quotes from your manuscript), or elaborate on this item by providing additional information not in the ms, or briefly explain why the item is not applicable/relevant for your study

All of the volunteers had at least a basic level of computer literacy and could use VR headsets and controllers after instruction.

4a-ii) Open vs. closed, web-based vs. face-to-face assessments:

Open vs. closed, web-based vs. face-to-face assessments: Mention how participants were recruited (online vs. offline), e.g., from an open access website or from a clinic, and clarify if this was a purely web-based trial, or there were face-to-face components (as part of the intervention or for assessment), i.e., to what degree got the study team to know the participant. In online-only trials, clarify if participants were quasi-anonymous and whether having multiple identities was possible or whether technical or logistical measures (e.g., cookies, email confirmation, phone calls) were used to detect/prevent these.

	1	2	3	4	5	
subitem not at all important	○	○	○	○	◉	essential

清除選取的項目

Does your paper address subitem 4a-ii? *

Copy and paste relevant sections from the manuscript (include quotes in quotation marks "like this" to indicate direct quotes from your manuscript), or elaborate on this item by providing additional information not in the ms, or briefly explain why the item is not applicable/relevant for your study

We used five different face-to-face assessments, including the Paas-CLS and NASA-TLX for subjectively estimating the cognitive load, the Milestone for assessing the learning outcomes, the AttrakDiff2 questionnaire and anonymous qualitative feedback for determining the learning experience. There were one objective estimate of cognitive load using the secondary-task performance and one measurement of cardiac autonomic function using the HRV monitoring.

你正在編輯自己的回覆內容。只要與他人共用這個網址，就能允許其他使用者編輯你的回覆內容。 填寫新的回覆

4a-iii) Information giving during recruitment

Information given during recruitment. Specify how participants were briefed for recruitment and in the informed consent procedures (e.g., publish the informed consent documentation as appendix, see also item X26), as this information may have an effect on user self-selection, user expectation and may also bias results.

	1	2	3	4	5	
subitem not at all important	○	○	○	○	◉	essential

清除選取的項目

Does your paper address subitem 4a-iii?

Copy and paste relevant sections from the manuscript (include quotes in quotation marks "like this" to indicate direct quotes from your manuscript), or elaborate on this item by providing additional information not in the ms, or briefly explain why the item is not applicable/relevant for your study

We informed the participants about the aims of the study and then obtained written informed consent from them.

4b) Settings and locations where the data were collected

Does your paper address CONSORT subitem 4b? *

Copy and paste relevant sections from the manuscript (include quotes in quotation marks "like this" to indicate direct quotes from your manuscript), or elaborate on this item by providing additional information not in the ms, or briefly explain why the item is not applicable/relevant for your study

We conducted a prospective, randomized controlled, pilot system-design study from 1 June 2018 to 30 October 2018 at an academic teaching hospital (Department of ORL-HNS, Linkou Chang Gung Memorial Hospital, Taoyuan, Taiwan).

你正在編輯自己的回覆內容。只要與他人共用這個網址，就能允許其他使用者編輯你的回覆內容。 填寫新的回覆

4b-i) Report if outcomes were (self-)assessed through online questionnaires

Clearly report if outcomes were (self-)assessed through online questionnaires (as common in web-based trials) or otherwise.

	1	2	3	4	5	
subitem not at all important	○	○	◉	○	○	essential

清除選取的項目

Does your paper address subitem 4b-i? *

Copy and paste relevant sections from the manuscript (include quotes in quotation marks "like this" to indicate direct quotes from your manuscript), or elaborate on this item by providing additional information not in the ms, or briefly explain why the item is not applicable/relevant for your study

No, we did not use oneline questionnaires in this study.

4b-ii) Report how institutional affiliations are displayed

Report how institutional affiliations are displayed to potential participants [on ehealth media], as affiliations with prestigious hospitals or universities may affect volunteer rates, use, and reactions with regards to an intervention.(Not a required item – describe only if this may bias results)

	1	2	3	4	5	
subitem not at all important	○	○	○	○	◉	essential

清除選取的項目

Does your paper address subitem 4b-ii?

Copy and paste relevant sections from the manuscript (include quotes in quotation marks "like this" to indicate direct quotes from your manuscript), or elaborate on this item by providing additional information not in the ms, or briefly explain why the item is not applicable/relevant for your study

The funding source had no role in the design and conduct of the study; collection, management, analysis, and interpretation of the data; preparation, review, or approval of the manuscript; and the decision to submit the manuscript for publication.

你正在編輯自己的回覆內容。只要與他人共用這個網址，就能允許其他使用者編輯你的回覆內容。 填寫新的回覆

5) The interventions for each group with sufficient details to allow replication, including how and when they were actually administered

5-i) Mention names, credential, affiliations of the developers, sponsors, and owners

Mention names, credential, affiliations of the developers, sponsors, and owners [6] (if authors/evaluators are owners or developer of the software, this needs to be declared in a “Conflict of interest” section or mentioned elsewhere in the manuscript).

	1	2	3	4	5	
subitem not at all important	○	○	○	◉	○	essential

清除選取的項目

Does your paper address subitem 5-i?

Copy and paste relevant sections from the manuscript (include quotes in quotation marks "like this" to indicate direct quotes from your manuscript), or elaborate on this item by providing additional information not in the ms, or briefly explain why the item is not applicable/relevant for your study

We produced two videos with different visual angles (360° and 120°) using PowerDirector software version 16 (Cyberlink Corp., New Taipei, Taiwan). Two senior investigators evaluated the videos and validated the learning materials. We then developed courseware with the same user interface for the 360° VR and 2-D videos using Unity 2017.3.1 Editor (Unity Technologies, San Francisco, CA, United States).

5-ii) Describe the history/development process

Describe the history/development process of the application and previous formative evaluations (e.g., focus groups, usability testing), as these will have an impact on adoption/use rates and help with interpreting results.

	1	2	3	4	5	
subitem not at all important	○	○	○	◉	○	essential

清除選取的項目

你正在編輯自己的回覆內容。只要與他人共用這個網址，就能允許其他使用者編輯你的回覆內容。 填寫新的回覆

Does your paper address subitem 5-ii?

Copy and paste relevant sections from the manuscript (include quotes in quotation marks "like this" to indicate direct quotes from your manuscript), or elaborate on this item by providing additional information not in the ms, or briefly explain why the item is not applicable/relevant for your study

We used analysis, design, development, implementation, and evaluation models [51] to design an effective instruction module for H&P, including essential knowledge and competence according to the guidelines of the American Board of Otolaryngology [7]. We used different working samples [52], including instructions for how to formulate problems, solution steps, and final solutions, to demonstrate step-by-step how to perform an H&P task in an outpatient setting. We also used self-explanation prompts [53] to encourage the learner to recognize links between the knowledge and skills they learned. We recorded a 10-min 360° video (4K resolution, 30 frames/s) with in-camera stitching, capturing 360° audio, and spherical stabilization using a 360° camera (Garmin VIRB 360, Garmin Ltd., Kansas City, MO, United States). We constructed the contents and scenario of this video according to a real clinical setting. The first portion of the video demonstrated skills of history taking under normal conditions and the second portion demonstrated skills of how to quickly perform a physical examination (Table 1). Subsequently, we produced two videos with different visual angles (360° and 120°) using PowerDirector software version 16 (Cyberlink Corp., New Taipei, Taiwan). Two senior investigators evaluated the videos and validated the learning materials. We then developed courseware with the same user interface for the 360° VR and 2-D videos using Unity 2017.3.1 Editor (Unity Technologies, San Francisco, CA, United States).

5-iii) Revisions and updating

Revisions and updating. Clearly mention the date and/or version number of the application/intervention (and comparator, if applicable) evaluated, or describe whether the intervention underwent major changes during the evaluation process, or whether the development and/or content was “frozen” during the trial. Describe dynamic components such as news feeds or changing content which may have an impact on the replicability of the intervention (for unexpected events see item 3b).

	1	2	3	4	5	
subitem not at all important	○	○	○	●	○	essential

清除選取的項目

你正在編輯自己的回覆內容。只要與他人共用這個網址，就能允許其他使用者編輯你的回覆內容。 填寫新的回覆

Does your paper address subitem 5-iii?

Copy and paste relevant sections from the manuscript (include quotes in quotation marks "like this" to indicate direct quotes from your manuscript), or elaborate on this item by providing additional information not in the ms, or briefly explain why the item is not applicable/relevant for your study

We used analysis, design, development, implementation, and evaluation models [51] to design an effective instruction module for H&P, including essential knowledge and competence according to the guidelines of the American Board of Otolaryngology [7]. We used different working samples [52], including instructions for how to formulate problems, solution steps, and final solutions, to demonstrate step-by-step how to perform an H&P task in an outpatient setting. We also used self-explanation prompts [53] to encourage the learner to recognize links between the knowledge and skills they learned. We recorded a 10-min 360° video (4K resolution, 30 frames/s) with in-camera stitching, capturing 360° audio, and spherical stabilization using a 360° camera (Garmin VIRB 360, Garmin Ltd., Kansas City, MO, United States). We constructed the contents and scenario of this video according to a real clinical setting. The first portion of the video demonstrated skills of history taking under normal conditions and the second portion demonstrated skills of how to quickly perform a physical examination (Table 1). Subsequently, we produced two videos with different visual angles (360° and 120°) using PowerDirector software version 16 (Cyberlink Corp., New Taipei, Taiwan). Two senior investigators evaluated the videos and validated the learning materials. We then developed courseware with the same user interface for the 360° VR and 2-D videos using Unity 2017.3.1 Editor (Unity Technologies, San Francisco, CA, United States).

5-iv) Quality assurance methods

Provide information on quality assurance methods to ensure accuracy and quality of information provided [1], if applicable.

	1	2	3	4	5	
subitem not at all important	○	○	○	◉	○	essential

清除選取的項目

Does your paper address subitem 5-iv?

Copy and paste relevant sections from the manuscript (include quotes in quotation marks "like this" to indicate direct quotes from your manuscript), or elaborate on this item by providing additional information not in the ms, or briefly explain why the item is not applicable/relevant for your study

Two senior investigators evaluated the videos and validated the learning materials.

你正在編輯自己的回覆內容。只要與他人共用這個網址，就能允許其他使用者編輯你的回覆內容。 填寫新的回覆

5-v) Ensure replicability by publishing the source code, and/or providing screenshots/screen-capture video, and/or providing flowcharts of the algorithms used

Ensure replicability by publishing the source code, and/or providing screenshots/screen-capture video, and/or providing flowcharts of the algorithms used. Replicability (i.e., other researchers should in principle be able to replicate the study) is a hallmark of scientific reporting.

	1	2	3	4	5	
subitem not at all important	○	○	○	○	◉	essential

清除選取的項目

Does your paper address subitem 5-v?

Copy and paste relevant sections from the manuscript (include quotes in quotation marks "like this" to indicate direct quotes from your manuscript), or elaborate on this item by providing additional information not in the ms, or briefly explain why the item is not applicable/relevant for your study

The 360° VR Video Module
This module was developed to arbitrarily review the immersive 3-D 360° VR video through a head-mounted display (Figure 2). The users were immersed in the 360° experience to learn the H&P skills.
The 2-D Video Module
The 2-D video was played in a fixed 120° focused field of view through the same head-mounted display (Figure 3). The users reviewed the instructional video as in a theater environment.

5-vi) Digital preservation

Digital preservation: Provide the URL of the application, but as the intervention is likely to change or disappear over the course of the years; also make sure the intervention is archived (Internet Archive, webcitation.org, and/or publishing the source code or screenshots/videos alongside the article). As pages behind login screens cannot be archived, consider creating demo pages which are accessible without login.

	1	2	3	4	5	
subitem not at all important	○	○	○	◉	○	essential

你正在編輯自己的回覆內容。只要與他人共用這個網址，就能允許其他使用者編輯你的回覆內容。

填寫新的回覆

Does your paper address subitem 5-vi?

Copy and paste relevant sections from the manuscript (include quotes in quotation marks "like this" to indicate direct quotes from your manuscript), or elaborate on this item by providing additional information not in the ms, or briefly explain why the item is not applicable/relevant for your study

Clinicaltrials.gov NCT03501641; https://clinicaltrials.gov/ct2/show/NCT03501641 (Archived by WebCite at http://www.webcitation.org/72f59ImWm)

5-vii) Access

Access: Describe how participants accessed the application, in what setting/context, if they had to pay (or were paid) or not, whether they had to be a member of specific group. If known, describe how participants obtained "access to the platform and Internet" [1]. To ensure access for editors/reviewers/readers, consider to provide a "backdoor" login account or demo mode for reviewers/readers to explore the application (also important for archiving purposes, see vi).

	1	2	3	4	5	
subitem not at all important	○	○	○	◉	○	essential

清除選取的項目

Does your paper address subitem 5-vii? *

Copy and paste relevant sections from the manuscript (include quotes in quotation marks "like this" to indicate direct quotes from your manuscript), or elaborate on this item by providing additional information not in the ms, or briefly explain why the item is not applicable/relevant for your study

We conducted a prospective, randomized controlled, pilot system-design study from 1 June 2018 to 30 October 2018 at an academic teaching hospital (Department of ORL-HNS, Linkou Chang Gung Memorial Hospital, Taoyuan, Taiwan). The Institutional Review Board of Chang Gung Medical Foundation approved this study (No: 201601821B0), and we conducted all procedures in compliance with the Declaration of Helsinki 1975. A convenience sampling approach was used, and 32 consecutive volunteers were recruited during the study period.

你正在編輯自己的回覆內容。只要與他人共用這個網址，就能允許其他使用者編輯你的回覆內容。 填寫新的回覆

5-viii) Mode of delivery, features/functionalities/components of the intervention and comparator, and the theoretical framework

Describe mode of delivery, features/functionalities/components of the intervention and comparator, and the theoretical framework [6] used to design them (instructional strategy [1], behaviour change techniques, persuasive features, etc., see e.g., [7, 8] for terminology). This includes an in-depth description of the content (including where it is coming from and who developed it) [1]," whether [and how] it is tailored to individual circumstances and allows users to track their progress and receive feedback" [6]. This also includes a description of communication delivery channels and – if computer-mediated communication is a component – whether communication was synchronous or asynchronous [6]. It also includes information on presentation strategies [1], including page design principles, average amount of text on pages, presence of hyperlinks to other resources, etc. [1].

	1	2	3	4	5	
subitem not at all important	○	○	○	◉	○	essential

清除選取的項目

Does your paper address subitem 5-viii? *

Copy and paste relevant sections from the manuscript (include quotes in quotation marks "like this" to indicate direct quotes from your manuscript), or elaborate on this item by providing additional information not in the ms, or briefly explain why the item is not applicable/relevant for your study

We used analysis, design, development, implementation, and evaluation models [51] to design an effective instruction module for H&P, including essential knowledge and competence according to the guidelines of the American Board of Otolaryngology [7]. We used different working samples [52], including instructions for how to formulate problems, solution steps, and final solutions, to demonstrate step-by-step how to perform an H&P task in an outpatient setting. We also used self-explanation prompts [53] to encourage the learner to recognize links between the knowledge and skills they learned.

5-ix) Describe use parameters

Describe use parameters (e.g., intended "doses" and optimal timing for use). Clarify what instructions or recommendations were given to the user, e.g., regarding timing, frequency, heaviness of use, if any, or was the intervention used ad libitum.

	1	2	3	4	5	
subitem not at all important	○	○	○	◉	○	essential

清除選取的項目

你正在編輯自己的回覆內容。只要與他人共用這個網址，就能允許其他使用者編輯你的回覆內容。 填寫新的回覆

Does your paper address subitem 5-ix?

Copy and paste relevant sections from the manuscript (include quotes in quotation marks "like this" to indicate direct quotes from your manuscript), or elaborate on this item by providing additional information not in the ms, or briefly explain why the item is not applicable/relevant for your study

After randomization, the participants reviewed their allocated video through a head-mounted display in the same space for 10 min. To reduce the effect of the head-mounted display on learning experience, both groups used the same VR device (Vive VR headset, HTC Corp., New Taipei, Taiwan).

5-x) Clarify the level of human involvement

Clarify the level of human involvement (care providers or health professionals, also technical assistance) in the e-intervention or as co-intervention (detail number and expertise of professionals involved, if any, as well as “type of assistance offered, the timing and frequency of the support, how it is initiated, and the medium by which the assistance is delivered”. It may be necessary to distinguish between the level of human involvement required for the trial, and the level of human involvement required for a routine application outside of a RCT setting (discuss under item 21 – generalizability).

	1	2	3	4	5	
subitem not at all important	○	○	○	●	○	essential

清除選取的項目

Does your paper address subitem 5-x?

Copy and paste relevant sections from the manuscript (include quotes in quotation marks "like this" to indicate direct quotes from your manuscript), or elaborate on this item by providing additional information not in the ms, or briefly explain why the item is not applicable/relevant for your study

To reduce the effect of the head-mounted display on learning experience, both groups used the same VR device (Vive VR headset, HTC Corp., New Taipei, Taiwan). We explained the functionality of the VR device to the participants before the intervention. In the 360° VR video group, the learners arbitrarily reviewed the instructor’s demonstrations and responses of standard patients and other medical staff from a first-person perspective in an immersive 360° environment (Figure 2). In the 2-D video group, the learners simply watched the instructor’s demonstrations from a third-person perspective in a theater environment (Figure 3). During the learning course, the participants watched the movie by themselves at a time of their choosing.

你正在編輯自己的回覆內容。只要與他人共用這個網址，就能允許其他使用者編輯你的回覆內容。 填寫新的回覆

5-xi) Report any prompts/reminders used

Report any prompts/reminders used: Clarify if there were prompts (letters, emails, phone calls, SMS) to use the application, what triggered them, frequency etc. It may be necessary to distinguish between the level of prompts/reminders required for the trial, and the level of prompts/reminders for a routine application outside of a RCT setting (discuss under item 21 – generalizability).

	1	2	3	4	5	
subitem not at all important	○	○	○	●	○	essential

清除選取的項目

Does your paper address subitem 5-xi? *

Copy and paste relevant sections from the manuscript (include quotes in quotation marks "like this" to indicate direct quotes from your manuscript), or elaborate on this item by providing additional information not in the ms, or briefly explain why the item is not applicable/relevant for your study

To reduce the effect of the head-mounted display on learning experience, both groups used the same VR device (Vive VR headset, HTC Corp., New Taipei, Taiwan). We explained the functionality of the VR device to the participants before the intervention. In the 360° VR video group, the learners arbitrarily reviewed the instructor's demonstrations and responses of standard patients and other medical staff from a first-person perspective in an immersive 360° environment (Figure 2). In the 2-D video group, the learners simply watched the instructor's demonstrations from a third-person perspective in a theater environment (Figure 3). During the learning course, the participants watched the movie by themselves at a time of their choosing.

5-xii) Describe any co-interventions (incl. training/support)

Describe any co-interventions (incl. training/support): Clearly state any interventions that are provided in addition to the targeted eHealth intervention, as ehealth intervention may not be designed as stand-alone intervention. This includes training sessions and support [1]. It may be necessary to distinguish between the level of training required for the trial, and the level of training for a routine application outside of a RCT setting (discuss under item 21 – generalizability.

	1	2	3	4	5	
subitem not at all important	○	○	○	●	○	essential

清除選取的項目

你正在編輯自己的回覆內容。只要與他人共用這個網址，就能允許其他使用者編輯你的回覆內容。 填寫新的回覆

Does your paper address subitem 5-xii? *

Copy and paste relevant sections from the manuscript (include quotes in quotation marks "like this" to indicate direct quotes from your manuscript), or elaborate on this item by providing additional information not in the ms, or briefly explain why the item is not applicable/relevant for your study

To reduce the effect of the head-mounted display on learning experience, both groups used the same VR device (Vive VR headset, HTC Corp., New Taipei, Taiwan). We explained the functionality of the VR device to the participants before the intervention. In the 360° VR video group, the learners arbitrarily reviewed the instructor's demonstrations and responses of standard patients and other medical staff from a first-person perspective in an immersive 360° environment (Figure 2). In the 2-D video group, the learners simply watched the instructor's demonstrations from a third-person perspective in a theater environment (Figure 3). During the learning course, the participants watched the movie by themselves at a time of their choosing.

6a) Completely defined pre-specified primary and secondary outcome measures, including how and when they were assessed

Does your paper address CONSORT subitem 6a? *

Copy and paste relevant sections from the manuscript (include quotes in quotation marks "like this" to indicate direct quotes from your manuscript), or elaborate on this item by providing additional information not in the ms, or briefly explain why the item is not applicable/relevant for your study

The primary outcome measure of this study was the Milestone level after completing the video learning module. The secondary outcomes were the AttrakDiff2 scales.

6a-i) Online questionnaires: describe if they were validated for online use and apply CHERRIES items to describe how the questionnaires were designed/deployed

If outcomes were obtained through online questionnaires, describe if they were validated for online use and apply CHERRIES items to describe how the questionnaires were designed/deployed [9].

	1	2	3	4	5	
subitem not at all important	○	○	○	◉	○	essential

清除選取的項目

你正在編輯自己的回覆內容。只要與他人共用這個網址，就能允許其他使用者編輯你的回覆內容。 填寫新的回覆

Does your paper address subitem 6a-i?

Copy and paste relevant sections from manuscript text

No, we did not use online questionnaire in this study.

6a-ii) Describe whether and how “use” (including intensity of use/dosage) was defined/measured/monitored

Describe whether and how “use” (including intensity of use/dosage) was defined/measured/monitored (logins, logfile analysis, etc.). Use/adoption metrics are important process outcomes that should be reported in any ehealth trial.

	1	2	3	4	5	
subitem not at all important	○	○	○	◉	○	essential

清除選取的項目

Does your paper address subitem 6a-ii?

Copy and paste relevant sections from manuscript text

During the learning course, the participants watched the movie by themselves at a time of their choosing.

6a-iii) Describe whether, how, and when qualitative feedback from participants was obtained

Describe whether, how, and when qualitative feedback from participants was obtained (e.g., through emails, feedback forms, interviews, focus groups).

	1	2	3	4	5	
subitem not at all important	○	○	○	◉	○	essential

清除選取的項目

你正在編輯自己的回覆內容。只要與他人共用這個網址，就能允許其他使用者編輯你的回覆內容。 填寫新的回覆

Does your paper address subitem 6a-iii?

Copy and paste relevant sections from manuscript text

Each participant in this study provided anonymous feedback about their experience of the module used immediate after the Milestone assessment.

6b) Any changes to trial outcomes after the trial commenced, with reasons

Does your paper address CONSORT subitem 6b? *

Copy and paste relevant sections from the manuscript (include quotes in quotation marks "like this" to indicate direct quotes from your manuscript), or elaborate on this item by providing additional information not in the ms, or briefly explain why the item is not applicable/relevant for your study

We did not change to trial outcomes after trial commenced.

7a) How sample size was determined

NPT: When applicable, details of whether and how the clustering by care provides or centers was addressed

7a-i) Describe whether and how expected attrition was taken into account when calculating the sample size

Describe whether and how expected attrition was taken into account when calculating the sample size.

	1	2	3	4	5	
subitem not at all important	○	○	○	○	◉	essential

清除選取的項目

你正在編輯自己的回覆內容。只要與他人共用這個網址，就能允許其他使用者編輯你的回覆內容。 填寫新的回覆

Does your paper address subitem 7a-i?

Copy and paste relevant sections from manuscript title (include quotes in quotation marks "like this" to indicate direct quotes from your manuscript), or elaborate on this item by providing additional information not in the ms, or briefly explain why the item is not applicable/relevant for your study

The sample size was estimated using primary outcome effects (the Milestone) based on a priori study (360° VR video: 3.1±0.7; 2-D video: 2.3±0.6). A two-tailed Wilcoxon-Mann-Whitney test was used to calculate a sample size of 16 in each group (normal parent distribution; calculated effect size, 1.23; type I error, 0.01; power, 70%). For a block size of 8, we decided to enroll a total of 32 students to show the difference in the Milestone level.

7b) When applicable, explanation of any interim analyses and stopping guidelines

Does your paper address CONSORT subitem 7b? *

Copy and paste relevant sections from the manuscript (include quotes in quotation marks "like this" to indicate direct quotes from your manuscript), or elaborate on this item by providing additional information not in the ms, or briefly explain why the item is not applicable/relevant for your study

No, this was a pilot study and we did not perform interim analyses setup stopping guidelines.

8a) Method used to generate the random allocation sequence

NPT: When applicable, how care providers were allocated to each trial group

Does your paper address CONSORT subitem 8a? *

Copy and paste relevant sections from the manuscript (include quotes in quotation marks "like this" to indicate direct quotes from your manuscript), or elaborate on this item by providing additional information not in the ms, or briefly explain why the item is not applicable/relevant for your study

"The Random Number Generator in IBM SPSS software (version 24; IBM, Armonk, NY, USA)" was used to create a list of random numbers for allocating the students, who were stratified by center with a 1:1 allocation using a fixed block size of 8 (Rv. Uniform [0, 1]) in both parallel subgroups.

你正在編輯自己的回覆內容。只要與他人共用這個網址，就能允許其他使用者編輯你的回覆內容。 填寫新的回覆

8b) Type of randomisation; details of any restriction (such as blocking and block size)

Does your paper address CONSORT subitem 8b? *

Copy and paste relevant sections from the manuscript (include quotes in quotation marks "like this" to indicate direct quotes from your manuscript), or elaborate on this item by providing additional information not in the ms, or briefly explain why the item is not applicable/relevant for your study

The Random Number Generator in IBM SPSS software (version 24; IBM, Armonk, NY, USA) was used to create a list of random numbers for allocating the students, who were stratified by center with a 1:1 allocation "using a fixed block size of 8" (Rv. Uniform [0, 1]) in both parallel subgroups.

9) Mechanism used to implement the random allocation sequence (such as sequentially numbered containers), describing any steps taken to conceal the sequence until interventions were assigned

Does your paper address CONSORT subitem 9? *

Copy and paste relevant sections from the manuscript (include quotes in quotation marks "like this" to indicate direct quotes from your manuscript), or elaborate on this item by providing additional information not in the ms, or briefly explain why the item is not applicable/relevant for your study

We concealed the allocation sequence before implementing the video module, and the module adhered to our computer-generated randomization protocol.

10) Who generated the random allocation sequence, who enrolled participants, and who assigned participants to interventions

Does your paper address CONSORT subitem 10? *

Copy and paste relevant sections from the manuscript (include quotes in quotation marks "like this" to indicate direct quotes from your manuscript), or elaborate on this item by providing additional information not in the ms, or briefly explain why the item is not applicable/relevant for your study

你正在編輯自己的回覆內容。只要與他人共用這個網址，就能允許其他使用者編輯你的回覆內容。

填寫新的回覆

11a) If done, who was blinded after assignment to interventions (for example, participants, care providers, those assessing outcomes) and how

NPT: Whether or not administering co-interventions were blinded to group assignment

11a-i) Specify who was blinded, and who wasn't

Specify who was blinded, and who wasn't. Usually, in web-based trials it is not possible to blind the participants [1, 3] (this should be clearly acknowledged), but it may be possible to blind outcome assessors, those doing data analysis or those administering co-interventions (if any).

	1	2	3	4	5	
subitem not at all important	○	○	◉	○	○	essential

清除選取的項目

Does your paper address subitem 11a-i? *

Copy and paste relevant sections from the manuscript (include quotes in quotation marks "like this" to indicate direct quotes from your manuscript), or elaborate on this item by providing additional information not in the ms, or briefly explain why the item is not applicable/relevant for your study

The participants were blinded to the purpose of the study during recruitment to minimize preparation bias. We concealed the allocation sequence before implementing the video module, and the module adhered to our computer-generated randomization protocol.

11a-ii) Discuss e.g., whether participants knew which intervention was the "intervention of interest" and which one was the "comparator"

Informed consent procedures (4a-ii) can create biases and certain expectations - discuss e.g., whether participants knew which intervention was the "intervention of interest" and which one was the "comparator".

	1	2	3	4	5	
subitem not at all important	○	○	○	◉	○	essential

清除選取的項目

你正在編輯自己的回覆內容。只要與他人共用這個網址，就能允許其他使用者編輯你的回覆內容。 填寫新的回覆

Does your paper address subitem 11a-ii?

Copy and paste relevant sections from the manuscript (include quotes in quotation marks "like this" to indicate direct quotes from your manuscript), or elaborate on this item by providing additional information not in the ms, or briefly explain why the item is not applicable/relevant for your study

In the 360° VR video group, the learners arbitrarily reviewed the instructor’s demonstrations and responses of standard patients and other medical staff from a first-person perspective in an immersive 360° environment (Figure 2). In the 2-D video group, the learners simply watched the instructor’s demonstrations from a third-person perspective in a theater environment (Figure 3).

11b) If relevant, description of the similarity of interventions

(this item is usually not relevant for ehealth trials as it refers to similarity of a placebo or sham intervention to a active medication/intervention)

Does your paper address CONSORT subitem 11b? *

Copy and paste relevant sections from the manuscript (include quotes in quotation marks "like this" to indicate direct quotes from your manuscript), or elaborate on this item by providing additional information not in the ms, or briefly explain why the item is not applicable/relevant for your study

Subsequently, we produced two videos with different visual angles (360° and 120°) using PowerDirector software version 16 (Cyberlink Corp., New Taipei, Taiwan). Two senior investigators evaluated the videos and validated the learning materials. We then developed courseware with the same user interface for the 360° VR and 2-D videos using Unity 2017.3.1 Editor (Unity Technologies, San Francisco, CA, United States).

12a) Statistical methods used to compare groups for primary and secondary outcomes

NPT: When applicable, details of whether and how the clustering by care providers or centers was addressed

你正在編輯自己的回覆內容。只要與他人共用這個網址，就能允許其他使用者編輯你的回覆內容。 填寫新的回覆

Does your paper address CONSORT subitem 12a? *

Copy and paste relevant sections from the manuscript (include quotes in quotation marks "like this" to indicate direct quotes from your manuscript), or elaborate on this item by providing additional information not in the ms, or briefly explain why the item is not applicable/relevant for your study

The D'Agostino-Pearson omnibus normality test showed that most of the continuous variables were non-normally distributed, and they were presented as median and interquartile range (IQR). Differences between groups were analyzed using the Wilcoxon signed-rank test, the Mann-Whitney U test, or Fisher's exact test as appropriate. Effect sizes were calculated using the Hodges-Lehmann method for the Mann-Whiney U test and Wilcoxon signed rank test, or odds ratios for Fisher's exact test. The Spearman correlation test was used to analyze relationships between variables of interest. The Bonferroni correction was used to adjust P-values because of the increased risk of a type I error when making multiple statistical tests at the same time [72]. Continuous variables were dichotomized using the median split. Variables of interest were analyzed for multivariate logistic regression models. All P-values were two-sided, and statistical significance was accepted at P<.01. Statistical analyses were performed using G*Power 3.1.9.2 software (Heinrich-Heine University, Dusseldorf, Germany), GraphPad Prism for Windows version 7.0 (GraphPad Software Inc., San Diego, CA, USA), and SPSS software version 25 (International Business Machines Corp., Armonk, NY, USA).

12a-i) Imputation techniques to deal with attrition / missing values

Imputation techniques to deal with attrition / missing values: Not all participants will use the intervention/comparator as intended and attrition is typically high in ehealth trials. Specify how participants who did not use the application or dropped out from the trial were treated in the statistical analysis (a complete case analysis is strongly discouraged, and simple imputation techniques such as LOCF may also be problematic [4]).

	1	2	3	4	5	
subitem not at all important	○	○	○	◉	○	essential

清除選取的項目

Does your paper address subitem 12a-i? *

Copy and paste relevant sections from the manuscript (include quotes in quotation marks "like this" to indicate direct quotes from your manuscript), or elaborate on this item by providing additional information not in the ms, or briefly explain why the item is not applicable/relevant for your study

After randomization, all participants (100%) received the intended intervention. There were no protocol deviations in this study.

你正在編輯自己的回覆內容。只要與他人共用這個網址，就能允許其他使用者編輯你的回覆內容。 填寫新的回覆

12b) Methods for additional analyses, such as subgroup analyses and adjusted analyses

Does your paper address CONSORT subitem 12b? *

Copy and paste relevant sections from the manuscript (include quotes in quotation marks "like this" to indicate direct quotes from your manuscript), or elaborate on this item by providing additional information not in the ms, or briefly explain why the item is not applicable/relevant for your study

Using multivariate logistic regression models, the 360° VR video module was independently associated with the Milestone level≥3 (exp B=15.0, 95% confidence interval [CI] 2.3−99.6, P=0.005); it was also independently related to the reaction time-10th min≥3.6 sec (exp B=18.8, 95% confidence interval [CI] 3.2−110.8, P=0.001). The significant relationship between Paas-CLS score≥6 and the RR-5−10 min≥0.779 ms persisted after adjustment for video module (exp B=15.0, 95% CI 2.3−99.6, P=0.005).
The relationships between the reaction time-5th min≥1.6 sec and RR-5−10 min≥0.779 ms (exp B=11.0, 95% CI 1.7−71.4, P=0.012) and the LF/HF ratio-5−10 min≥1.43 and hedonic stimulation scale≥2.0 (exp B=0.14, 95% CI 0.03−0.68, P=0.015) were no more statistically significant after adjustment for video module.

X26) REB/IRB Approval and Ethical Considerations [recommended as subheading under "Methods"] (not a CONSORT item)

X26-i) Comment on ethics committee approval

	1	2	3	4	5	
subitem not at all important	○	○	○	○	◉	essential

清除選取的項目

你正在編輯自己的回覆內容。只要與他人共用這個網址，就能允許其他使用者編輯你的回覆內容。 填寫新的回覆

Does your paper address subitem X26-i?

Copy and paste relevant sections from the manuscript (include quotes in quotation marks "like this" to indicate direct quotes from your manuscript), or elaborate on this item by providing additional information not in the ms, or briefly explain why the item is not applicable/relevant for your study

The Institutional Review Board of Chang Gung Medical Foundation approved this study (No: 201601821B0), and we conducted all procedures in compliance with the Declaration of Helsinki 1975.

x26-ii) Outline informed consent procedures

Outline informed consent procedures e.g., if consent was obtained offline or online (how? Checkbox, etc.?), and what information was provided (see 4a-ii). See [6] for some items to be included in informed consent documents.

	1	2	3	4	5	
subitem not at all important	○	○	◉	○	○	essential

清除選取的項目

Does your paper address subitem X26-ii?

Copy and paste relevant sections from the manuscript (include quotes in quotation marks "like this" to indicate direct quotes from your manuscript), or elaborate on this item by providing additional information not in the ms, or briefly explain why the item is not applicable/relevant for your study

We informed the participants about the aims of the study and then obtained written informed consent from them.

X26-iii) Safety and security procedures

Safety and security procedures, incl. privacy considerations, and any steps taken to reduce the likelihood or detection of harm (e.g., education and training, availability of a hotline)

	1	2	3	4	5	
subitem not at all important	○	○	○	◉	○	essential

清除選取的項目

你正在編輯自己的回覆內容。只要與他人共用這個網址，就能允許其他使用者編輯你的回覆內容。

填寫新的回覆

Does your paper address subitem X26-iii?

Copy and paste relevant sections from the manuscript (include quotes in quotation marks "like this" to indicate direct quotes from your manuscript), or elaborate on this item by providing additional information not in the ms, or briefly explain why the item is not applicable/relevant for your study

To reduce the effect of the head-mounted display on learning experience, both groups used the same VR device (Vive VR headset, HTC Corp., New Taipei, Taiwan).

RESULTS

13a) For each group, the numbers of participants who were randomly assigned, received intended treatment, and were analysed for the primary outcome

NPT: The number of care providers or centers performing the intervention in each group and the number of patients treated by each care provider in each center

Does your paper address CONSORT subitem 13a? *

Copy and paste relevant sections from the manuscript (include quotes in quotation marks "like this" to indicate direct quotes from your manuscript), or elaborate on this item by providing additional information not in the ms, or briefly explain why the item is not applicable/relevant for your study

Thirty-two volunteers (20 males [62%] and 12 females [38%]; median age 24 years, IQR 23–25 years; 3 field-dependence [9%] and 29 field-independence [91%]) were recruited. Table 2 summarizes the variables of interest for the overall study cohort. As expected, there were no significant differences in age, sex, or cognitive style between the 360° VR video and 2-D video groups at baseline.

13b) For each group, losses and exclusions after randomisation, together with reasons

你正在編輯自己的回覆內容。只要與他人共用這個網址，就能允許其他使用者編輯你的回覆內容。

填寫新的回覆

Does your paper address CONSORT subitem 13b? (NOTE: Preferably, this is shown in a CONSORT flow diagram) *

Copy and paste relevant sections from the manuscript (include quotes in quotation marks "like this" to indicate direct quotes from your manuscript), or elaborate on this item by providing additional information not in the ms, or briefly explain why the item is not applicable/relevant for your study

After randomization, all participants (100%) received the intended intervention. There were no protocol deviations in this study.

13b-i) Attrition diagram

Strongly recommended: An attrition diagram (e.g., proportion of participants still logging in or using the intervention/comparator in each group plotted over time, similar to a survival curve) or other figures or tables demonstrating usage/dose/engagement.

	1	2	3	4	5	
subitem not at all important	○	○	○	◉	○	essential

清除選取的項目

Does your paper address subitem 13b-i?

Copy and paste relevant sections from the manuscript or cite the figure number if applicable (include quotes in quotation marks "like this" to indicate direct quotes from your manuscript), or elaborate on this item by providing additional information not in the ms, or briefly explain why the item is not applicable/relevant for your study

We did not evaluate an attrition diagrram due to a pilot study design.

14a) Dates defining the periods of recruitment and follow-up

你正在編輯自己的回覆內容。只要與他人共用這個網址，就能允許其他使用者編輯你的回覆內容。

填寫新的回覆

Does your paper address CONSORT subitem 14a? *

Copy and paste relevant sections from the manuscript (include quotes in quotation marks "like this" to indicate direct quotes from your manuscript), or elaborate on this item by providing additional information not in the ms, or briefly explain why the item is not applicable/relevant for your study

We conducted a prospective, randomized controlled, pilot system-design study from 1 June 2018 to 30 October 2018 at an academic teaching hospital (Department of ORL-HNS, Linkou Chang Gung Memorial Hospital, Taoyuan, Taiwan).

14a-i) Indicate if critical “secular events” fell into the study period

Indicate if critical “secular events” fell into the study period, e.g., significant changes in Internet resources available or “changes in computer hardware or Internet delivery resources”

	1	2	3	4	5	
subitem not at all important	○	○	○	●	○	essential

清除選取的項目

Does your paper address subitem 14a-i?

Copy and paste relevant sections from the manuscript (include quotes in quotation marks "like this" to indicate direct quotes from your manuscript), or elaborate on this item by providing additional information not in the ms, or briefly explain why the item is not applicable/relevant for your study

No, we did not find secular events that fell into the study period.

14b) Why the trial ended or was stopped (early)

Does your paper address CONSORT subitem 14b? *

Copy and paste relevant sections from the manuscript (include quotes in quotation marks "like this" to indicate direct quotes from your manuscript), or elaborate on this item by providing additional information not in the ms, or briefly explain why the item is not applicable/relevant for your study

The trial ended because the sample size was large enough.

你正在編輯自己的回覆內容。只要與他人共用這個網址，就能允許其他使用者編輯你的回覆內容。 填寫新的回覆

15) A table showing baseline demographic and clinical characteristics for each group

NPT: When applicable, a description of care providers (case volume, qualification, expertise, etc.) and centers (volume) in each group

Does your paper address CONSORT subitem 15? *

Copy and paste relevant sections from the manuscript (include quotes in quotation marks "like this" to indicate direct quotes from your manuscript), or elaborate on this item by providing additional information not in the ms, or briefly explain why the item is not applicable/relevant for your study

Table 2. Demographics and cognitive style.

15-i) Report demographics associated with digital divide issues

In ehealth trials it is particularly important to report demographics associated with digital divide issues, such as age, education, gender, social-economic status, computer/Internet/ehealth literacy of the participants, if known.

	1	2	3	4	5	
subitem not at all important	○	○	○	◉	○	essential

清除選取的項目

Does your paper address subitem 15-i? *

Copy and paste relevant sections from the manuscript (include quotes in quotation marks "like this" to indicate direct quotes from your manuscript), or elaborate on this item by providing additional information not in the ms, or briefly explain why the item is not applicable/relevant for your study

As expected, there were no significant differences in age, sex, or cognitive style between the 360° VR video and 2-D video groups at baseline.

16) For each group, number of participants (denominator) included in each analysis and whether the analysis was by original assigned groups

你正在編輯自己的回覆內容。只要與他人共用這個網址，就能允許其他使用者編輯你的回覆內容。 填寫新的回覆

16-i) Report multiple “denominators” and provide definitions

Report multiple “denominators” and provide definitions: Report N’s (and effect sizes) “across a range of study participation [and use] thresholds” [1], e.g., N exposed, N consented, N used more than x times, N used more than y weeks, N participants “used” the intervention/comparator at specific pre-defined time points of interest (in absolute and relative numbers per group). Always clearly define “use” of the intervention.

	1	2	3	4	5	
subitem not at all important	○	○	○	●	○	essential

清除選取的項目

Does your paper address subitem 16-i? *

Copy and paste relevant sections from the manuscript (include quotes in quotation marks "like this" to indicate direct quotes from your manuscript), or elaborate on this item by providing additional information not in the ms, or briefly explain why the item is not applicable/relevant for your study

After randomization, all participants (100%) received the intended intervention. There were no protocol deviations in this study.

16-ii) Primary analysis should be intent-to-treat

Primary analysis should be intent-to-treat, secondary analyses could include comparing only “users”, with the appropriate caveats that this is no longer a randomized sample (see 18-i).

	1	2	3	4	5	
subitem not at all important	○	○	○	●	○	essential

清除選取的項目

你正在編輯自己的回覆內容。只要與他人共用這個網址，就能允許其他使用者編輯你的回覆內容。 填寫新的回覆

Does your paper address subitem 16-ii?

Copy and paste relevant sections from the manuscript (include quotes in quotation marks "like this" to indicate direct quotes from your manuscript), or elaborate on this item by providing additional information not in the ms, or briefly explain why the item is not applicable/relevant for your study

Overall, the participants had a significantly higher Milestone level (median level 3; IQR 2–4) than the reference value of "1" (P<.001) after 10 min of video instruction. Although the Milestone level of the 360° VR video group (median level 3; IQR 3–4) was higher than that of the 2-D video group (median level 2; IQR 2–3), the difference did not reach a statistical significance (effect size=1 [0–1]; P=.02) (Figure 4, lower).

17a) For each primary and secondary outcome, results for each group, and the estimated effect size and its precision (such as 95% confidence interval)

Does your paper address CONSORT subitem 17a? *

Copy and paste relevant sections from the manuscript (include quotes in quotation marks "like this" to indicate direct quotes from your manuscript), or elaborate on this item by providing additional information not in the ms, or briefly explain why the item is not applicable/relevant for your study

Tables 2-4 demonstrate the estimated effective size and its precision.

17a-i) Presentation of process outcomes such as metrics of use and intensity of use

In addition to primary/secondary (clinical) outcomes, the presentation of process outcomes such as metrics of use and intensity of use (dose, exposure) and their operational definitions is critical. This does not only refer to metrics of attrition (13-b) (often a binary variable), but also to more continuous exposure metrics such as "average session length". These must be accompanied by a technical description how a metric like a "session" is defined (e.g., timeout after idle time) [1] (report under item 6a).

	1	2	3	4	5	
subitem not at all important	○	○	○	●	○	essential

清除選取的項目

你正在編輯自己的回覆內容。只要與他人共用這個網址，就能允許其他使用者編輯你的回覆內容。 填寫新的回覆

Does your paper address subitem 17a-i?

Copy and paste relevant sections from the manuscript (include quotes in quotation marks "like this" to indicate direct quotes from your manuscript), or elaborate on this item by providing additional information not in the ms, or briefly explain why the item is not applicable/relevant for your study

After randomization, all participants (100%) received the intended intervention. There were no protocol deviations in this study.

17b) For binary outcomes, presentation of both absolute and relative effect sizes is recommended

Does your paper address CONSORT subitem 17b? *

Copy and paste relevant sections from the manuscript (include quotes in quotation marks "like this" to indicate direct quotes from your manuscript), or elaborate on this item by providing additional information not in the ms, or briefly explain why the item is not applicable/relevant for your study

Table 2 demonstrates the estimated effective size and its precision for binary outcomes.

18) Results of any other analyses performed, including subgroup analyses and adjusted analyses, distinguishing pre-specified from exploratory

Does your paper address CONSORT subitem 18? *

Copy and paste relevant sections from the manuscript (include quotes in quotation marks "like this" to indicate direct quotes from your manuscript), or elaborate on this item by providing additional information not in the ms, or briefly explain why the item is not applicable/relevant for your study

Using multivariate logistic regression models, the 360° VR video module was independently associated with the Milestone level≥3 (exp B=15.0, 95% confidence interval [CI] 2.3–99.6, P=0.005); it was also independently related to the reaction time-10th min≥3.6 sec (exp B=18.8, 95% confidence interval [CI] 3.2–110.8, P=0.001). The significant relationship between Paas-CLS score≥6 and the RR-5–10 min≥0.779 ms persisted after adjustment for video module (exp B=15.0, 95% CI 2.3–99.6, P=0.005).

你正在編輯自己的回覆內容。只要與他人共用這個網址，就能允許其他使用者編輯你的回覆內容。 填寫新的回覆

18-i) Subgroup analysis of comparing only users

A subgroup analysis of comparing only users is not uncommon in ehealth trials, but if done, it must be stressed that this is a self-selected sample and no longer an unbiased sample from a randomized trial (see 16-iii).

	1	2	3	4	5	
subitem not at all important	○	○	○	◉	○	essential

清除選取的項目

Does your paper address subitem 18-i?

Copy and paste relevant sections from the manuscript (include quotes in quotation marks "like this" to indicate direct quotes from your manuscript), or elaborate on this item by providing additional information not in the ms, or briefly explain why the item is not applicable/relevant for your study

No, we did not perform such subgroup analysis of comparing only users.

19) All important harms or unintended effects in each group

(for specific guidance see CONSORT for harms)

Does your paper address CONSORT subitem 19? *

Copy and paste relevant sections from the manuscript (include quotes in quotation marks "like this" to indicate direct quotes from your manuscript), or elaborate on this item by providing additional information not in the ms, or briefly explain why the item is not applicable/relevant for your study

Faults
However, three (19%) participants of the 360° VR video group reported that it caused 'mild VR dizziness.' Notably, the 2-D video learners said that the module was 'tedious' (n=3 [19%]) and that there was 'no interaction' (n=1 [6%]).

你正在編輯自己的回覆內容。只要與他人共用這個網址，就能允許其他使用者編輯你的回覆內容。

填寫新的回覆

19-i) Include privacy breaches, technical problems

Include privacy breaches, technical problems. This does not only include physical "harm" to participants, but also incidents such as perceived or real privacy breaches [1], technical problems, and other unexpected/unintended incidents. "Unintended effects" also includes unintended positive effects [2].

	1	2	3	4	5	
subitem not at all important	○	○	◉	○	○	essential

清除選取的項目

Does your paper address subitem 19-i?

Copy and paste relevant sections from the manuscript (include quotes in quotation marks "like this" to indicate direct quotes from your manuscript), or elaborate on this item by providing additional information not in the ms, or briefly explain why the item is not applicable/relevant for your study

This study did not include privacy breaches, technical problems.

19-ii) Include qualitative feedback from participants or observations from staff/researchers

Include qualitative feedback from participants or observations from staff/researchers, if available, on strengths and shortcomings of the application, especially if they point to unintended/unexpected effects or uses. This includes (if available) reasons for why people did or did not use the application as intended by the developers.

	1	2	3	4	5	
subitem not at all important	○	○	○	◉	○	essential

清除選取的項目

你正在編輯自己的回覆內容。只要與他人共用這個網址，就能允許其他使用者編輯你的回覆內容。 填寫新的回覆

Does your paper address subitem 19-ii?

Copy and paste relevant sections from the manuscript (include quotes in quotation marks "like this" to indicate direct quotes from your manuscript), or elaborate on this item by providing additional information not in the ms, or briefly explain why the item is not applicable/relevant for your study

Benefits
The 360° VR video group reported that the 360° VR video module was 'fun to learn' (n=3 [19%]) and 'good for physical examinations' (n=1 [6%]). Qualitative feedback from the 2-D video group emphasized that they found the 2-D video module 'easy to follow' (n=2 [13%]) and 'highly efficient' (n=1 [6%]).
Faults
However, three (19%) participants of the 360° VR video group reported that it caused 'mild VR dizziness.' Notably, the 2-D video learners said that the module was 'tedious' (n=3 [19%]) and that there was 'no interaction' (n=1 [6%]).

DISCUSSION

22) Interpretation consistent with results, balancing benefits and harms, and considering other relevant evidence

NPT: In addition, take into account the choice of the comparator, lack of or partial blinding, and unequal expertise of care providers or centers in each group

22-i) Restate study questions and summarize the answers suggested by the data, starting with primary outcomes and process outcomes (use)

Restate study questions and summarize the answers suggested by the data, starting with primary outcomes and process outcomes (use).

	1	2	3	4	5	
subitem not at all important	○	○	○	○	◉	essential

清除選取的項目

你正在編輯自己的回覆內容。只要與他人共用這個網址，就能允許其他使用者編輯你的回覆內容。 填寫新的回覆

Does your paper address subitem 22-i? *

Copy and paste relevant sections from the manuscript (include quotes in quotation marks "like this" to indicate direct quotes from your manuscript), or elaborate on this item by providing additional information not in the ms, or briefly explain why the item is not applicable/relevant for your study

This study's main findings highlighted the complexities of cognitive load estimations and HRV indexes inherent in developing and evaluating 360° VR and 2D video learning. Our results showed that video learning resulted in a higher total cognitive load and mental demand and prolonged the reaction time-10th min; however, video learning also enhanced a Milestone level of H&P skills with positive learning experiences. Using the same head-mounted display, both the 360° VR and 2D videos were efficient learning methods with positive learning experiences for novice learners. Notably, the 360° VR video learners demonstrated a higher Milestone level of H&P in actual patients with SDB than the 2D video learners. Although both video modules produced comparable total cognitive load and subscales, the 360° VR video learners had a more prolonged reaction time-10th min than the 2D video learners.
Interestingly, in the second portion of the video, the 360° VR video learners had a longer reaction time than the baseline data. These findings suggested that the immersive 360° VR video could reduce secondary-task performance without increasing subjective cognitive loads. Furthermore, the elevated LF/HF ratio-0–5 min correlated with a reduced hedonic stimulation scale. Combined with qualitative feedback, we found that the students seemed to consume more cognitive resources to fit the immersive 360° VR video than the 2D video learning. This preliminary study suggested that the immersive 360° VR video might lead to a better Milestone level than the 2-D video, which seemed to be not related to cognitive load estimates and HRV indexes in novice learners.

22-ii) Highlight unanswered new questions, suggest future research

Highlight unanswered new questions, suggest future research.

	1	2	3	4	5	
subitem not at all important	○	○	○	◉	○	essential

清除選取的項目

你正在編輯自己的回覆內容。只要與他人共用這個網址，就能允許其他使用者編輯你的回覆內容。

填寫新的回覆

Does your paper address subitem 22-ii?

Copy and paste relevant sections from the manuscript (include quotes in quotation marks "like this" to indicate direct quotes from your manuscript), or elaborate on this item by providing additional information not in the ms, or briefly explain why the item is not applicable/relevant for your study

Accordingly, further studies with a larger sample size with the extended competence spectrum and learning outcome assessments are warranted to confirm our results and inferences.

20) Trial limitations, addressing sources of potential bias, imprecision, and, if relevant, multiplicity of analyses

20-i) Typical limitations in ehealth trials

Typical limitations in ehealth trials: Participants in ehealth trials are rarely blinded. Ehealth trials often look at a multiplicity of outcomes, increasing risk for a Type I error. Discuss biases due to non-use of the intervention/usability issues, biases through informed consent procedures, unexpected events.

	1	2	3	4	5	
subitem not at all important	○	○	○	○	◉	essential

清除選取的項目

Does your paper address subitem 20-i? *

Copy and paste relevant sections from the manuscript (include quotes in quotation marks "like this" to indicate direct quotes from your manuscript), or elaborate on this item by providing additional information not in the ms, or briefly explain why the item is not applicable/relevant for your study

This study used quantitative and qualitative measures and methods to evaluate different video learning materials and showed its potential clinical applicability for learning H&P skills. However, several limitations should be addressed. First, this study needs external validation due to the small sample size. Although H&P is one of the essential training topics for undergraduate medical students, our outcomes may not be generalizable to junior residents who already have basic knowledge, skills, and attitudes. Second, assessments of the learner's performance in the workplace, such as the Mini Clinical Evaluation Exercise, were not included in this study. Third, there were several significant associations between variables of interest in this study. However, the potential relationships between video module, cognitive load, and HRV might be underestimated using such stringent criteria for

你正在編輯自己的回覆內容。只要與他人共用這個網址，就能允許其他使用者編輯你的回覆內容。

填寫新的回覆

21) Generalisability (external validity, applicability) of the trial findings

NPT: External validity of the trial findings according to the intervention, comparators, patients, and care providers or centers involved in the trial

21-i) Generalizability to other populations

Generalizability to other populations: In particular, discuss generalizability to a general Internet population, outside of a RCT setting, and general patient population, including applicability of the study results for other organizations

	1	2	3	4	5	
subitem not at all important	○	○	○	◉	○	essential

清除選取的項目

Does your paper address subitem 21-i?

Copy and paste relevant sections from the manuscript (include quotes in quotation marks "like this" to indicate direct quotes from your manuscript), or elaborate on this item by providing additional information not in the ms, or briefly explain why the item is not applicable/relevant for your study

First, this study needs external validation due to the small sample size. Although H&P is one of the essential training topics for undergraduate medical students, our outcomes may not be generalizable to junior residents who already have basic knowledge, skills, and attitudes.

21-ii) Discuss if there were elements in the RCT that would be different in a routine application setting

Discuss if there were elements in the RCT that would be different in a routine application setting (e.g., prompts/reminders, more human involvement, training sessions or other co-interventions) and what impact the omission of these elements could have on use, adoption, or outcomes if the intervention is applied outside of a RCT setting.

	1	2	3	4	5	
subitem not at all important	○	○	◉	○	○	essential

清除選取的項目

你正在編輯自己的回覆內容。只要與他人共用這個網址，就能允許其他使用者編輯你的回覆內容。 填寫新的回覆

Does your paper address subitem 21-ii?

Copy and paste relevant sections from the manuscript (include quotes in quotation marks "like this" to indicate direct quotes from your manuscript), or elaborate on this item by providing additional information not in the ms, or briefly explain why the item is not applicable/relevant for your study

No, there were no elements that would be different in a routine application setting.

OTHER INFORMATION

23) Registration number and name of trial registry

Does your paper address CONSORT subitem 23? *

Copy and paste relevant sections from the manuscript (include quotes in quotation marks "like this" to indicate direct quotes from your manuscript), or elaborate on this item by providing additional information not in the ms, or briefly explain why the item is not applicable/relevant for your study

Clinicaltrials.gov NCT03501641; https://clinicaltrials.gov/ct2/show/NCT03501641 (Archived by WebCite at http://www.webcitation.org/72f59ImWm)

24) Where the full trial protocol can be accessed, if available

Does your paper address CONSORT subitem 24? *

Cite a Multimedia Appendix, other reference, or copy and paste relevant sections from the manuscript (include quotes in quotation marks "like this" to indicate direct quotes from your manuscript), or elaborate on this item by providing additional information not in the ms, or briefly explain why the item is not applicable/relevant for your study

Figure 1 shows the study flowchart following the CONSORT 2010 guidelines [50].

25) Sources of funding and other support (such as supply of drugs), role of funders

你正在編輯自己的回覆內容。只要與他人共用這個網址，就能允許其他使用者編輯你的回覆內容。 填寫新的回覆

Does your paper address CONSORT subitem 25? *

Copy and paste relevant sections from the manuscript (include quotes in quotation marks "like this" to indicate direct quotes from your manuscript), or elaborate on this item by providing additional information not in the ms, or briefly explain why the item is not applicable/relevant for your study

This work was financially supported by grants from the Ministry of Science and Technology, Taiwan, R.O.C. (106-2511-S-182A-003-MY2 and 108-2511-H-182A-001) and grants from the Chang Gung Medical Foundation, Taiwan, R.O.C. (CMRPG3G1381−3 and CMRPG3G1931).

X27) Conflicts of Interest (not a CONSORT item)

X27-i) State the relation of the study team towards the system being evaluated

In addition to the usual declaration of interests (financial or otherwise), also state the relation of the study team towards the system being evaluated, i.e., state if the authors/evaluators are distinct from or identical with the developers/sponsors of the intervention.

	1	2	3	4	5	
subitem not at all important	○	○	○	◉	○	essential

清除選取的項目

Does your paper address subitem X27-i?

Copy and paste relevant sections from the manuscript (include quotes in quotation marks "like this" to indicate direct quotes from your manuscript), or elaborate on this item by providing additional information not in the ms, or briefly explain why the item is not applicable/relevant for your study

The authors/evaluators are identical with the developers of the intervention.

About the CONSORT EHEALTH checklist

你正在編輯自己的回覆內容。只要與他人共用這個網址，就能允許其他使用者編輯你的回覆內容。 填寫新的回覆

As a result of using this checklist, did you make changes in your manuscript? *

- ○ yes, major changes
- ◉ yes, minor changes
- ○ no

What were the most important changes you made as a result of using this checklist?

The title and methods and results.

How much time did you spend on going through the checklist INCLUDING making changes in your manuscript *

I spent 5 hourspm gpomg through this checklist

As a result of using this checklist, do you think your manuscript has improved? *

- ◉ yes
- ○ no
- ○ 其他:

你正在編輯自己的回覆內容。只要與他人共用這個網址，就能允許其他使用者編輯你的回覆內容。 填寫新的回覆

Would you like to become involved in the CONSORT EHEALTH group?

This would involve for example becoming involved in participating in a workshop and writing an "Explanation and Elaboration" document

- ○ yes
- ◉ no
- ○ 其他：

清除選取的項目

Any other comments or questions on CONSORT EHEALTH

I have no comments or questions.

STOP - Save this form as PDF before you click submit

To generate a record that you filled in this form, we recommend to generate a PDF of this page (on a Mac, simply select "print" and then select "print as PDF") before you submit it.

When you submit your (revised) paper to JMIR, please upload the PDF as supplementary file.

Don't worry if some text in the textboxes is cut off, as we still have the complete information in our database. Thank you!

Final step: Click submit !

Click submit so we have your answers in our database!

提交

請勿利用 Google 表單送出密碼。

Google 並未認可或建立這項內容。 檢舉濫用情形 - 服務條款 - 隱私權政策

Google 表單

你正在編輯自己的回覆內容。只要與他人共用這個網址，就能允許其他使用者編輯你的回覆內容。

填寫新的回覆
